# All-Trans Retinoic Acid and Curcumin Exhibit Hormesis or Synergistic Anticancer Effects in U87 Glioblastoma Cells: Defining the Proteome Accompanying Synergism

**DOI:** 10.3390/ijms27177795

**Published:** 2026-08-31

**Authors:** Ceyda Sönmez, Meric A. Altinoz, Aleyna Baltacıoğlu, Büşra Ergün, Aysel Özpınar

**Affiliations:** 1 Department of Biochemistry and Molecular Biology, Graduate School of Health Sciences, Acibadem Mehmet Ali Aydinlar University, Istanbul 34752, Türkiye; ceyda.sonmez@live.acibadem.edu.tr (C.S.); adilmeric.altinoz@saglik.gov.tr (M.A.A.); aleyna.baltacioglu@live.acibadem.edu.tr (A.B.); busra.ergun@live.acibadem.edu.tr (B.E.); 2 Department of Public Health, Kagithane District Health Directorate, Turkish Ministry of Health, Istanbul 34408, Türkiye; 3 Department of Medical Biochemistry, School of Medicine, Acibadem Mehmet Ali Aydinlar University, Istanbul 34752, Türkiye

**Keywords:** glioblastoma, all-trans retinoic acid, curcumin, proliferation, migration, xCELLigence, apoptosis, flow cytometry, proteome

## Abstract

Persistently poor glioblastoma (GBM) survival necessitates better elucidation of tumor drug responses. After observing that low curcumin and all-trans retinoic acid (ATRA) doses stimulated cell proliferation and counteracted each other’s high-dose antiproliferative effects in U87 GBM cells, drug influences on cell growth, migration, and death and the antiproliferative interaction proteome were further studied. Cell proliferation and migration were assessed by xCELLigence Real-Time Cell Analysis (RTCA). Cell death was defined using flow cytometry. Drug interactions were determined with CompuSyn software (version 1.0). Liquid Chromatography–Tandem Mass Spectrometry (LC-MS/MS), High-Performance Liquid Chromatography (HPLC), and SequestHT software (version 1.4) were utilized for peptide generation and identification. ATRA at high doses inhibited cell growth and migration more efficiently. Curcumin was more proliferative and antagonistic against anti-growth effects at low doses. Migration inhibition and apoptosis occurred synergistically at the highest drug doses. ATRA influenced the proteome more remarkably, reducing Transforming Growth Factor Beta Induced (TGFBI), Phosphoglycerate Dehydrogenase (PHGDH), tenascin, and Sequestosome 1 (SQSTM1). These effects were alleviated by curcumin, except for SQSTM1. Uveal Autoantigen With Coiled-Coil Domains And Ankyrin Repeats (UACA) and Sad1 And UNC84 Domain Containing 2 (SUN2) were increased by ATRA and curcumin, and to a lesser extent by the combination. Hexokinase 2 (HXK2) was increased by curcumin and the combination. Heme Oxygenase 1 (HMOX1) was depleted by the combination, but not by the single agents. SQSTM1 and HMOX1 reductions may mediate anticancer synergism, while the remaining changes may indicate ongoing hormetic pathways not reflected in cell counts.

## 1. Introduction

Glioblastoma (GBM), also known as glioblastoma multiforme, is a highly aggressive central nervous system malignancy classified as WHO (World Health Organization) grade 4 [1]. Despite surgery combined with radiotherapy and temozolomide (TMZ) chemotherapy during and after treatment, median survival remains approximately 12–15 months. The five-year survival rate is still below 10%, reflecting only limited progress despite decades of research [2]. Countless molecules have been identified that individually contribute to resistance mechanisms in malignancies with poor prognosis, but these molecules may actually belong to distinct sets of resistance mechanisms despite interacting with one another [3]. Therefore, classifying cancer resistance behavior into core mechanisms can help discover novel ways to simultaneously manipulate large numbers of molecules for treatment, among which hormesis can be proposed as one of the foremost means of cellular resilience. Hormesis is defined as an accommodative dose response wherein biotic or abiotic stressors provide protective influences that improve the performance of cells and organisms at low doses while acting harmfully and leading to worse fitness outcomes, accelerated aging, and death at high doses [4]. The term hormesis, from the Greek word “eagerness,” was first suggested in 1943 by Southam and Erhlich, who observed that fungal cell metabolism and growth improved after treatment with red cedar (*Juniperus virginiana*) extracts containing the mitotic spindle poison podophyllotoxin, which itself and its derivatives are cytotoxic anticancer agents [5,6,7]. In parallel, agents tested for antineoplastic effects may act hormetically at low doses, while their cytotoxicity emerges at higher doses. Although hormesis mechanisms are well documented, fewer studies have examined the pathways underlying the antineoplastic synergism of agents that show mutual antagonism at low doses but synergize at high doses. This is most likely due to scientists’ preference for reporting synergistic interactions between anticancer agents. The literature on the role of hormesis in glial neoplasms remains limited, but emerging evidence suggests that hormesis substantially shapes their drug responses [8,9,10].

One approach to cancer management is to drive terminal differentiation of malignant cells, leading to their reduced resilience, as terminal differentiation may lower cancer cells’ death thresholds [11,12]. For instance, GBM cells undergo simultaneous terminal differentiation and death when blocked at the internal ribosome entry site (IRES)-mediated translation, a specialized protein-synthesis mode that malignant cells depend on to resist adverse microenvironmental conditions [13]. The first clinical implication of cancer differentiation therapy involves the use of all-trans retinoic acid (ATRA) in acute promyelocytic leukemia treatment [12]. ATRA is a biologically active metabolite of vitamin A (retinol), produced by aldehyde dehydrogenases, and retinoids (retinol derivatives) are key embryonic morphogens that regulate cell differentiation during development [14,15]. Retinoids are primarily involved in neuroectoderm morphogenesis, and central nervous system (CNS) malignancies are of neuroectodermal origin [14,16]. In parallel, high ATRA doses promote differentiation and reduce stemness and O^6^-methylguanine-DNA methyltransferase (MGMT) expression in GBM cells [17]. Despite limited clinical studies on retinoid therapies in GBM, observations of 13-cis-retinoic acid-naphthalene triazole and ATRA treatment in patients with high-grade glial tumors revealed objective responses, which seem to be more prominent with the latter [18,19]. A lesser-known feature of ATRA is its tumorigenic effect under specific conditions, which appears particularly relevant to glial tumors and will be contextualized in the discussion.

Curcumin is a phenolic pigment of the diarylheptanoid class, giving *Curcuma longa* its yellow-orange color, and is used as a phytomedicine. Despite research demonstrating its potent antioxidant, anti-inflammatory, and anticancer activities, low aqueous solubility, rapid metabolism and clearance limit the clinical translation of these effects—challenges that could be overcome with nanoformulations [20]. The antitumor efficacy of curcumin closely relates to its apoptotic actions, which involve the repression of anti-apoptotic NF-κB and PI3K/Akt/mTOR pathways [21]. Curcumin induction of cancer cell apoptosis also involves increasing reactive oxygen species (ROS)-induced DNA damage, downregulating the anti-apoptotic proteins IAP, Bcl-2, Bcl-xL, and Mcl-1, and upregulating or augmenting the efficacy of the pro-apoptotic proteins Bim, p53, and TRAIL, as shown in several cancers, including GBM [21]. While nanodelivery strategies may overcome curcumin’s pharmacokinetic limitations, optimizing its cancer treatment application still requires clearer elucidation of the pathways underlying its antineoplastic activity. Curcumin’s anticancer mechanisms may overlap with or differ from those of ATRA, and their interactions during co-application could augment or limit antitumor activity.

The main reason for combining ATRA with curcumin is that both agents exert significant differentiation-inducing effects on so-called stem-like glioma cells (SLGCs) or glioma-initiating cells (GICs) despite possessing completely unrelated chemical structures [22,23]. In a study consisting of both in vitro and in vivo experiments using live microscopy in a cranial window setting, ATRA significantly suppressed the growth and invasion of SLGCs, reduced the release of proangiogenic cytokines, and augmented the proapoptotic effects of cisplatin, 1,3-bis(2-chloroethyl)-1-nitrosourea (BCNU) or carmustine, and radiation [22]. Similarly, curcumin exerts profound differentiation-inducing effects on GICs, accompanied by intense autophagy leading to cytotoxic effects in cell culture and the inhibition of the tumor-forming capacities of GICs implanted into mouse brains [23]. Recent evidence supporting the ATRA and curcumin combination in GBM treatment comprises observations that nanoparticle-mediated ATRA and curcumin co-delivery achieved greater inhibition of GBM cell proliferation and migration, accompanied by enhanced apoptosis, compared with either agent alone [20].

Most previous studies have focused primarily on phenotypic outcomes or on a limited number of predefined molecular targets using conventional targeted approaches. However, such methods may not fully capture the broad and interconnected molecular alterations induced by combination treatments. Mass spectrometry-based proteomics enables the large-scale characterization of treatment-associated changes in protein expression and provides insight into the biological processes, signaling pathways, and protein interaction networks involved in the cellular response. Therefore, proteomic investigation of the ATRA–curcumin combination may contribute to a more comprehensive understanding of its anticancer activity in glioblastoma and help identify the molecular mechanisms underlying the observed therapeutic response. Thus, we aimed to detail the cell growth patterns accompanying opposite interactions, as well as the cell death, migration features, and proteomic profile associated with synergistic anticancer effects. The implications of the results may extend beyond these two agents and improve our understanding of GBM drug responses.

## 2. Results

### 2.1. ATRA and Curcumin Effects on Cell Proliferation

**Figure 1:**
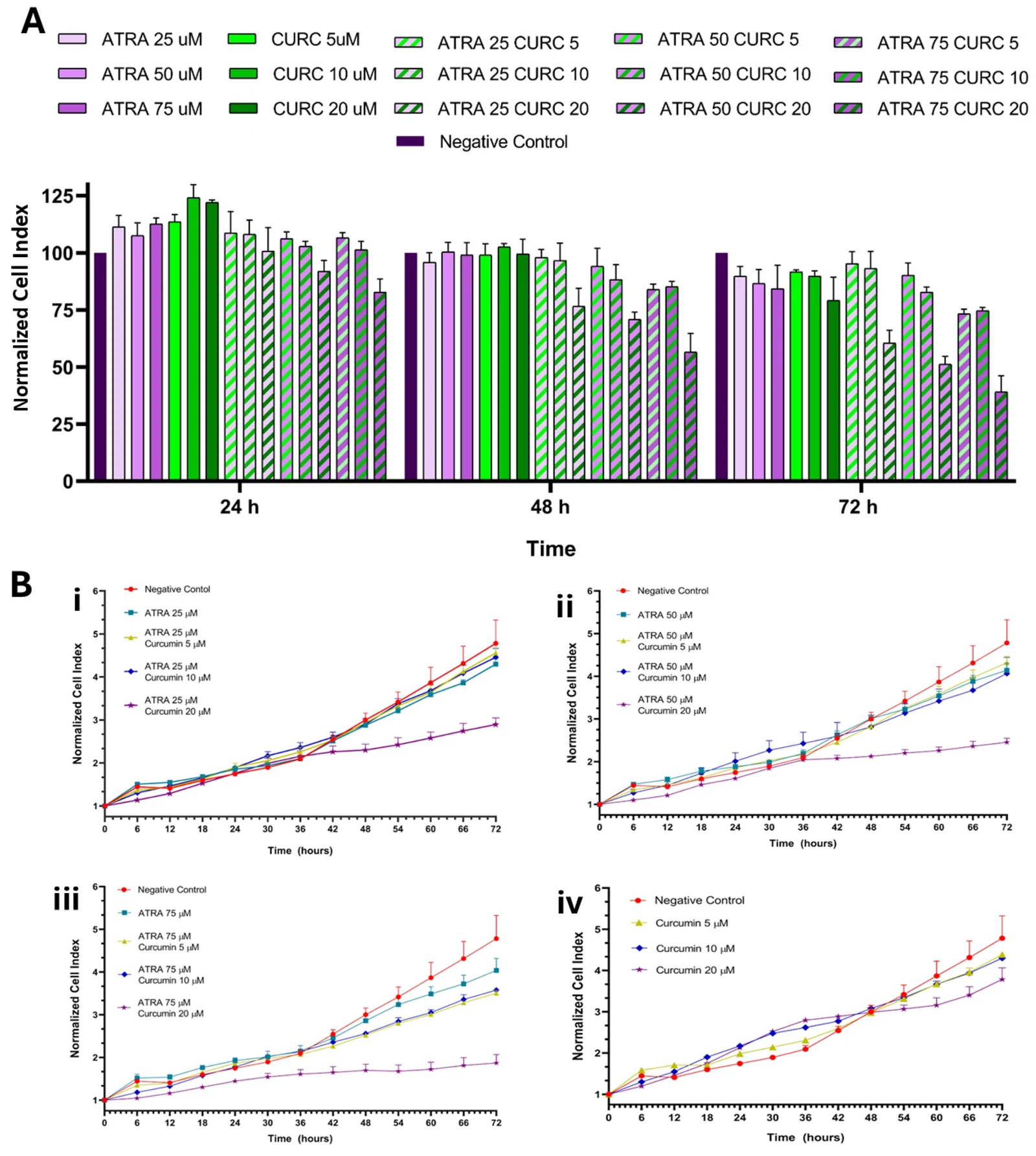
All-trans retinoic acid (ATRA) and curcumin (CURC) effects on cell proliferation. (**A**) The bar graph represents the entire experimental group at 24, 48, and 72 h. (**B**) The line graph depicts the time-dependent effects of ATRA at 25 µM (**i**), 50 µM (**ii**), and 75 µM (**iii**), each combined with varying curcumin doses (5, 10, and 20 µM), and the time-dependent effects of curcumin (**iv**). Abbreviations: ATRA: All-trans Retinoic Acid; CURC: Curcumin.

**Figure 2:**
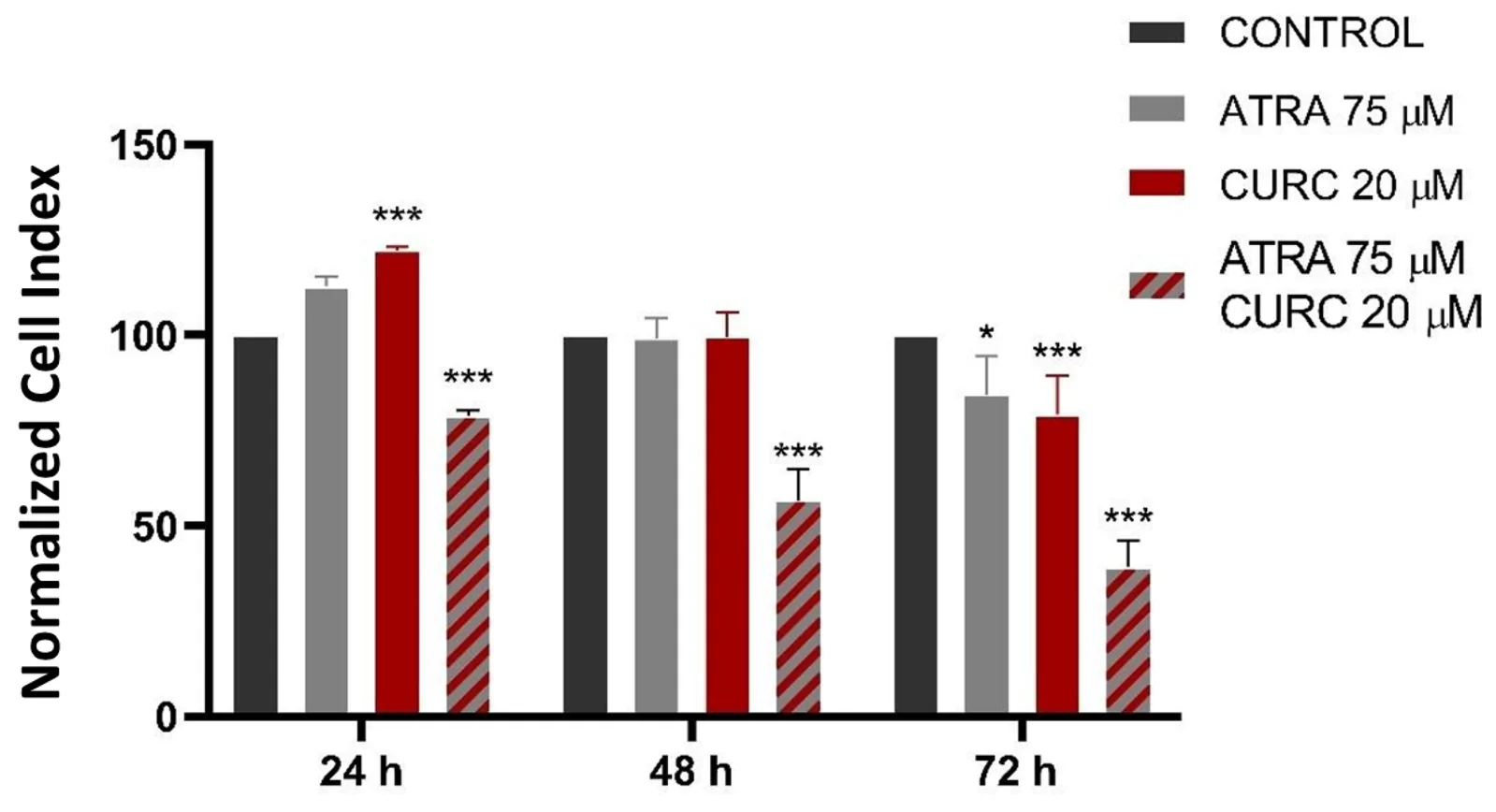
Effects of ATRA (75 μM), curcumin (20 μM), and their combination on cell proliferation. Notably, single agents had relatively subtle effects on proliferation at 48 h, whereas the combination decreased cell growth beginning at 24 h and continued to decline progressively through 72 h (*p* = 0.033, marked by *; *p* < 0.001, marked by ***). Abbreviations: ATRA: All-trans Retinoic Acid; CURC: Curcumin.

Figure 1 and Figure 2 depict the effects of ATRA, curcumin, and their combination on cell proliferation, with the latter prepared to more clearly demonstrate a strongly significant synergism at 72 h. Table 1 shows the Fraction of Cells Affected (FCA) and Combination Index (CI) values that indicate drug interaction types (i.e., synergistic, additive, or antagonistic). As indicated in the methodology, drug-induced cell proliferation at early time points precluded performing statistical synergy tests due to CompuSyn software limitations. Therefore, the data presented on drug interactions are limited to the effects observed at 72 h. Regarding cell proliferation, a notable feature was that both ATRA and curcumin, as single agents, promoted cell growth at 24 h, with the latter acting more pronouncedly. In combination groups, curcumin at 5 and 10 µM showed strong antagonism of ATRA antineoplasticity at 25 µM, and even the lowest curcumin dose (5 µM) inhibited ATRA antineoplasticity at 50 µM, with these effects reflected in Table 1 by CI values exceeding 10, indicating antagonistic interactions. The highest ATRA dose was more effective at suppressing cell growth than the highest curcumin dose and interacted synergistically to suppress cell growth when applied at 50 µM or higher, with this synergistic effect markedly intensifying over time. Curcumin at 10 µM synergistically and significantly augmented growth inhibition mediated by ATRA at 50 or 75 µM (*p* < 0.01), and at 20 µM, it synergistically enhanced the antiproliferative effects of all applied ATRA dosages, with even higher significance (*p* < 0.001). The CI value for the ATRA (75 µM) and curcumin (20 µM) combination was below 0.1, suggesting a robust synergism at 72 h. Hence, further experiments on cell death and proteomics were conducted with the ATRA 75 µM + curcumin 20 µM combination.

### 2.2. ATRA and Curcumin Effects on Cell Migration

**Figure 3:**
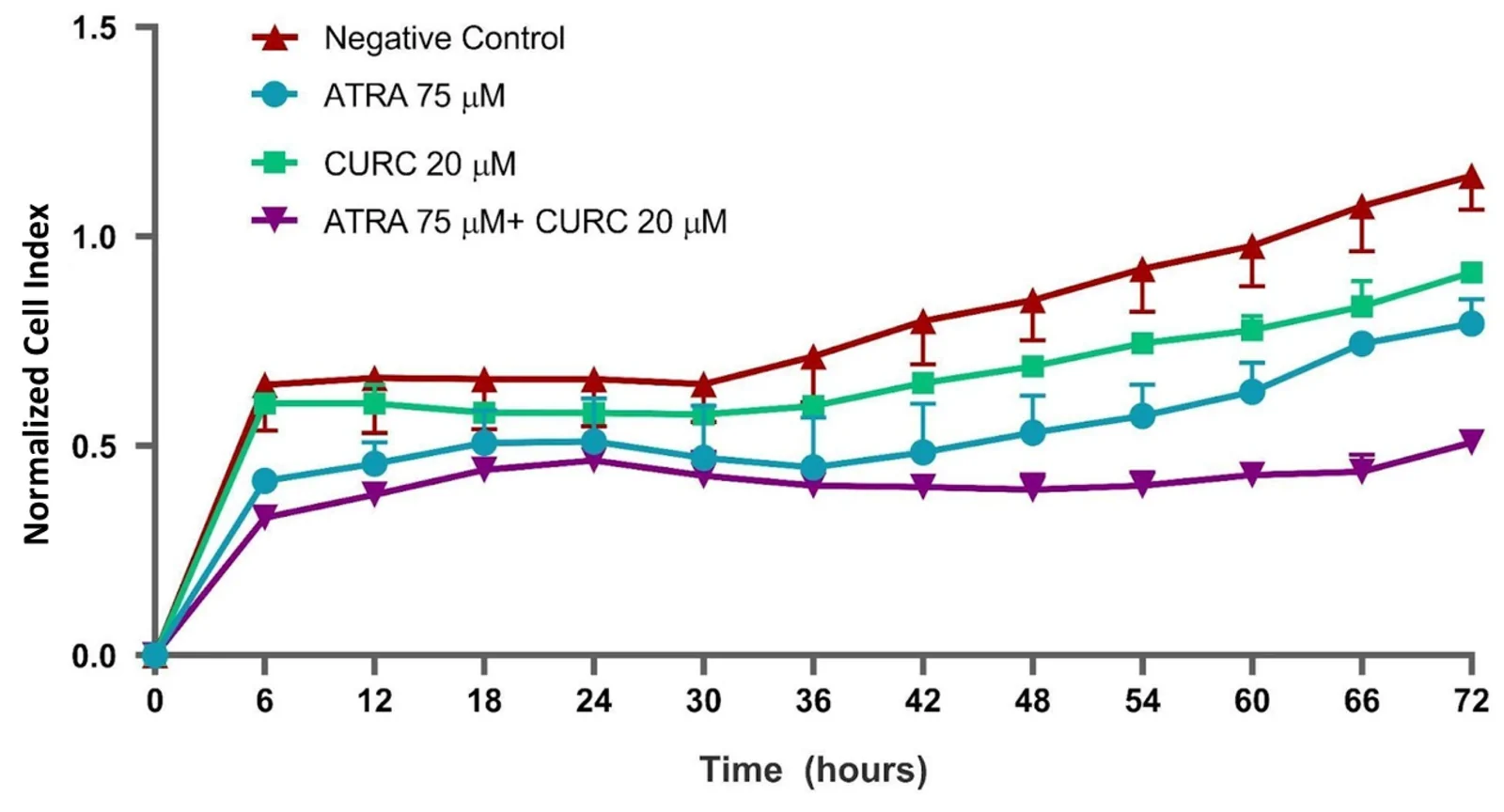
Effects of ATRA (75 μM), curcumin (20 μM), and their combination on U87 GBM cell migration over 72 h. Abbreviations: ATRA: All-trans Retinoic Acid; CURC: Curcumin.

Figure 3 demonstrates the effects of ATRA and curcumin on cell migration. While control group cells migrated continuously until 72 h, all treatments suppressed migration, with combination treatments exerting more pronounced effects. Until 12 h, the normalized cell index (NCI) values in the curcumin group closely paralleled those in the control group, whereas only minor differences existed in the ATRA and combination groups. From 36 h onwards, the inhibitory profiles of the single agents became more evident. ATRA suppressed cell migration between 1 and 14 h (*p* < 0.05) and again after 34 h, with an NCI value stabilizing at approximately 0.55 by 72 h. Curcumin remained less potent than ATRA and exhibited a delayed yet detectable effect, becoming significant from 39 h onwards, with an NCI value reaching approximately 0.65 at 72 h. The combination group exerted the most consistent and prominent inhibition of cell migration compared to the other groups, starting as early as 1 h (*p* < 0.05). With the combined treatment, NCI values plateaued earlier and remained markedly lower, reaching only about 0.35 at 72 h, with statistically significant effects demonstrated in Supplementary Table S1.

### 2.3. ATRA and Curcumin Effects on Cell Death

**Figure 4:**
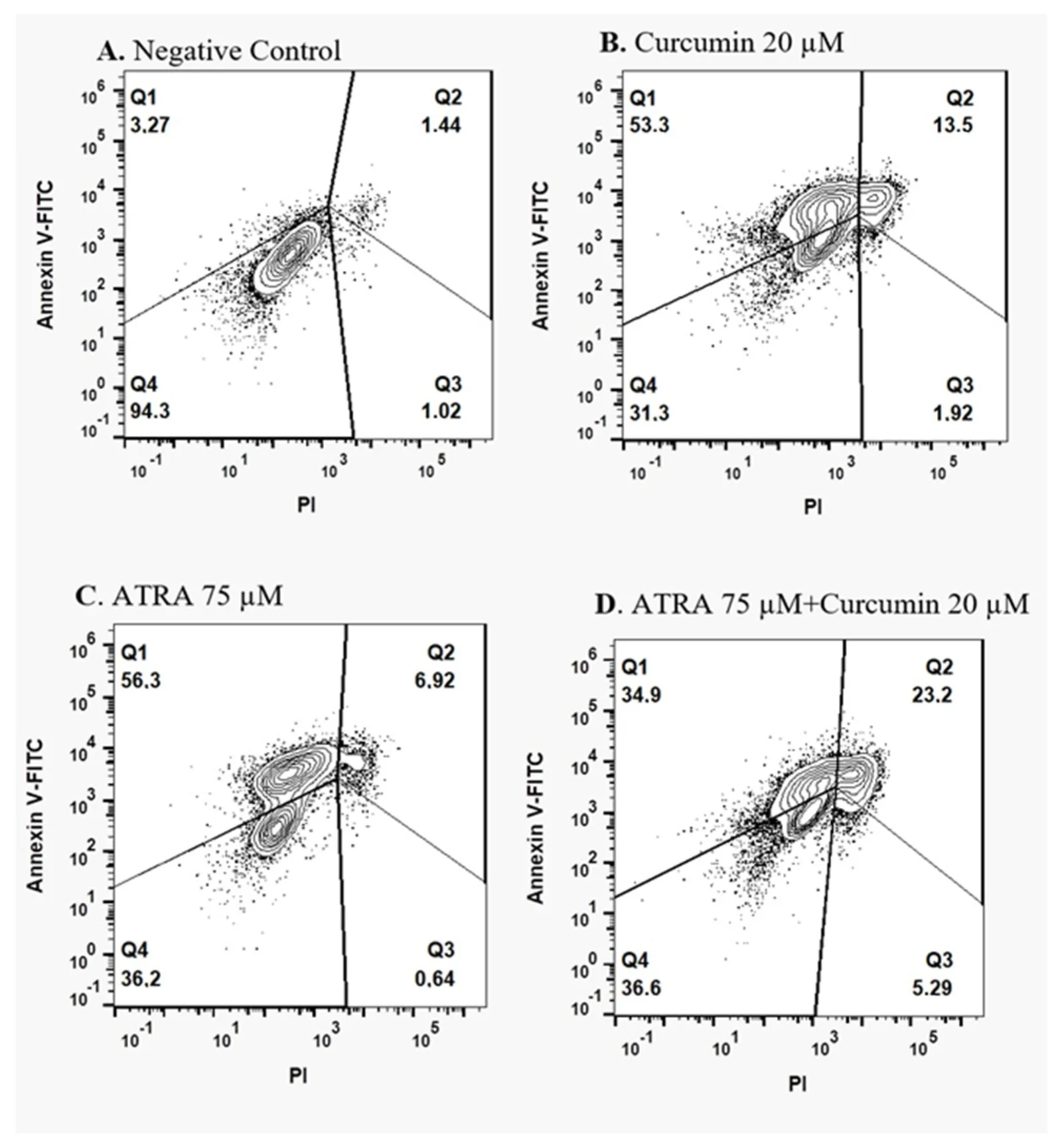
Effects of ATRA and curcumin on U87 GBM cell death. (**A**) Negative Control; (**B**): Curcumin (20 µM); (**C**): ATRA (75 µM); (**D**): Combination. Abbreviations: ATRA: All-trans Retinoic Acid.

Figure 4 shows the drugs’ single or combined influences on cell death patterns. Flow cytometric analysis of U87 GBM cells stained with Annexin V-FITC and propidium iodide (PI) at 72 h revealed that 94.3% of the cells in the control group remained alive, with no signs of apoptosis. Cells treated with ATRA or curcumin alone exhibited markedly elevated levels of early apoptosis, with rates of 53.3% and 56.3%, respectively. Yet, the rates of late apoptosis were lower in single-drug treatments, at 13.5% and 6.92% for ATRA and curcumin, respectively. Moreover, each agent alone caused very low necrosis rates, suggesting a tendency to stimulate apoptosis rather than to induce nonspecific necrotic cell death. A substantial decrease in cell viability in the combination group was accompanied by a marked increase in late apoptosis to 23.2%. This finding suggests that cells progressed through the apoptosis stages, culminating in loss of membrane integrity, as evidenced by the elevation in the necrosis rate.

### 2.4. Proteins Showing Differential Expression Between Treatment Groups

**Figure 5:**
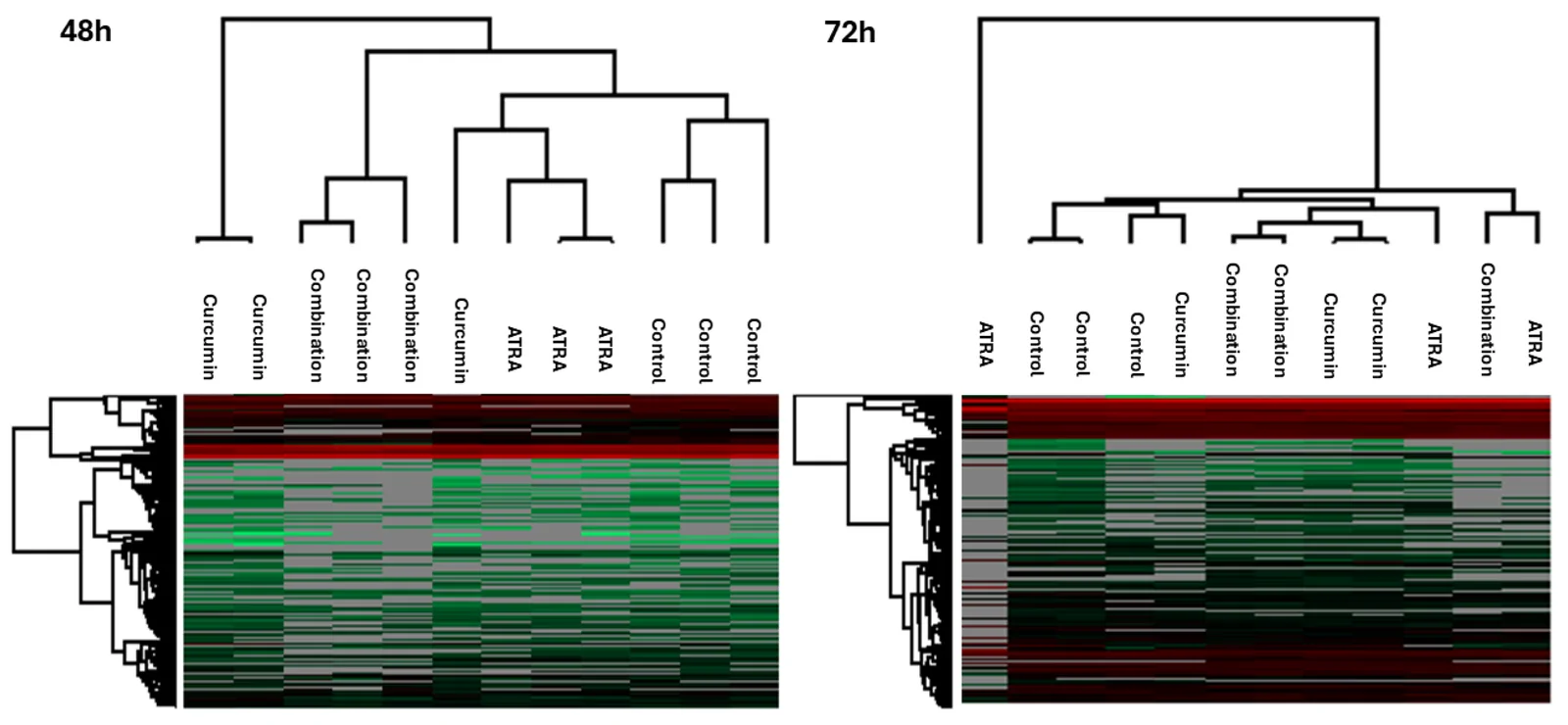
Hierarchical clustering of differentially expressed proteins across treatments. Abbreviations: ATRA: All-trans Retinoic Acid.

Hierarchical clustering analysis was performed to identify similarities and differences in protein expression among the experimental groups. Quantitative analysis across all samples identified 1457 and 1723 proteins at 48 h and 72 h, respectively. The resulting dendrogram structure and color distribution showed proteomic separations between the groups (Figure 5). According to the 48-h data, the proteins in the combination group formed distinct clusters, whereas one of the curcumin groups was clustered with the ATRA group. At 72 h, two of the three from the ATRA and curcumin groups clustered with the combination group, whereas one from the ATRA group exhibited an expression profile distinct from the remaining groups.

**Figure 6:**
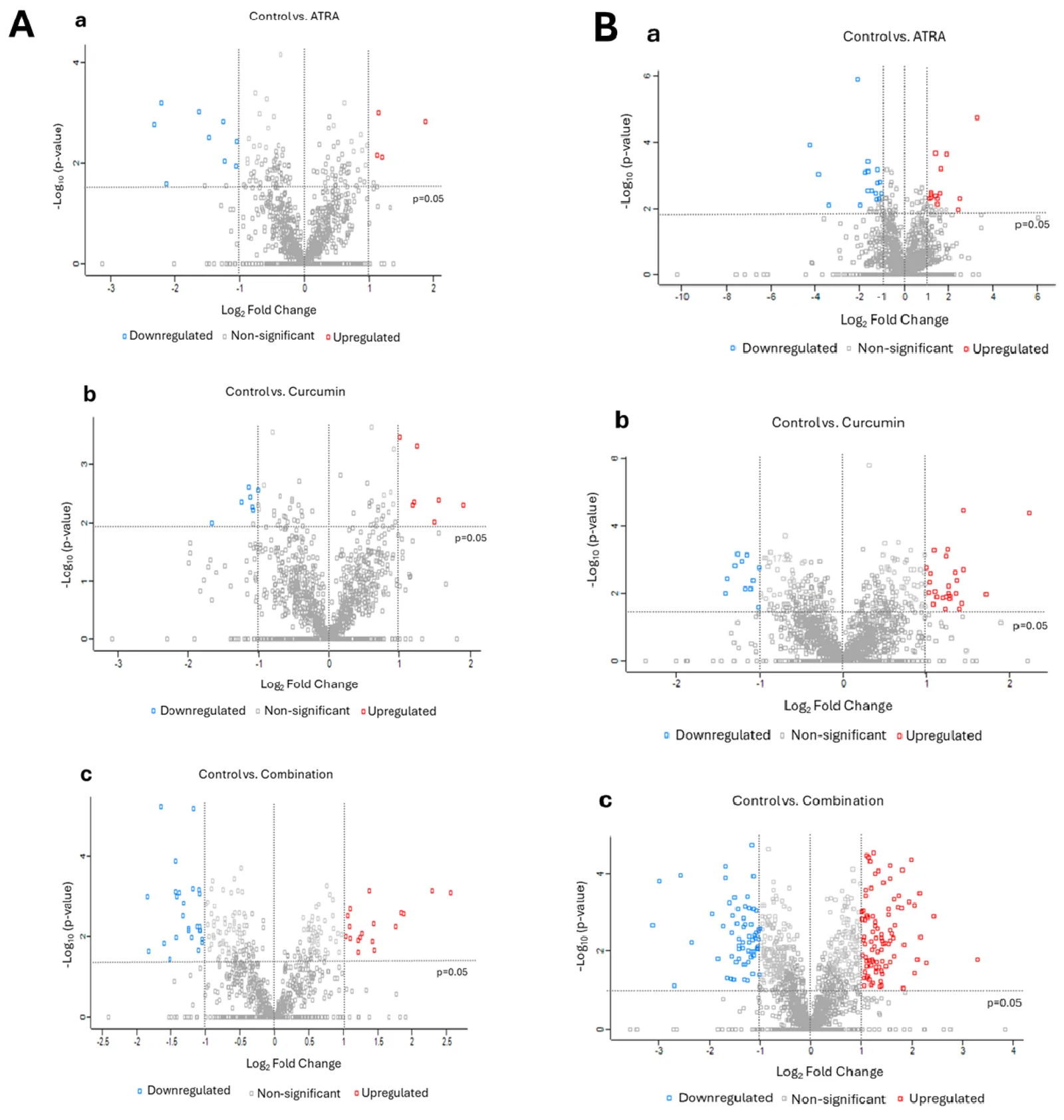
Volcano plots showing proteins differentially expressed between (**A**) 48 h and (**B**) 72 h treatment groups. Volcano plots depict the distribution of proteins based on the log_2_ fold change (x-axis) and −log_10_(*p*-value) (y-axis), comparing experimental groups to the control group. Each point represents individual protein. (**a**) Comparison between control and ATRA-treated cells; (**b**) control and curcumin-treated cells; (**c**) control and combined (ATRA + curcumin) treatment groups. Proteins significantly downregulated in treatment groups compared to control are shown in blue, while upregulated proteins are shown in red. Gray points indicate non-significant changes. Significance threshold was set based on *t*-test *p*-values (*p* < 0.05) and fold change cutoffs (|log_2_FC| > 1).

Statistically significant protein expression changes (*p* < 0.05) across different treatments are presented in volcano plots (Figure 6). Compared with the control, 71 proteins were differentially expressed at 48 h, including 13, 14, and 44 in the ATRA, curcumin, and combination groups, respectively. At the same interval, 9, 7, and 26 proteins were downregulated, and 4, 7, and 18 proteins were upregulated in the ATRA, curcumin, and combination groups, respectively. Compared with the control, 224 proteins were differentially expressed at 72 h, including 31, 37, and 156 in the ATRA, curcumin, and combination groups, respectively. At the same interval, 17, 11, and 63 proteins were downregulated, and 14, 26, and 93 proteins were upregulated in the ATRA, curcumin, and combination groups, respectively. These findings suggest that ATRA and curcumin alone influenced a relatively limited protein repertoire, whereas their combination modified a broader spectrum of protein expression.

### 2.5. Molecular Functions and Pathways of Differentially Expressed Proteins

**Figure 7:**
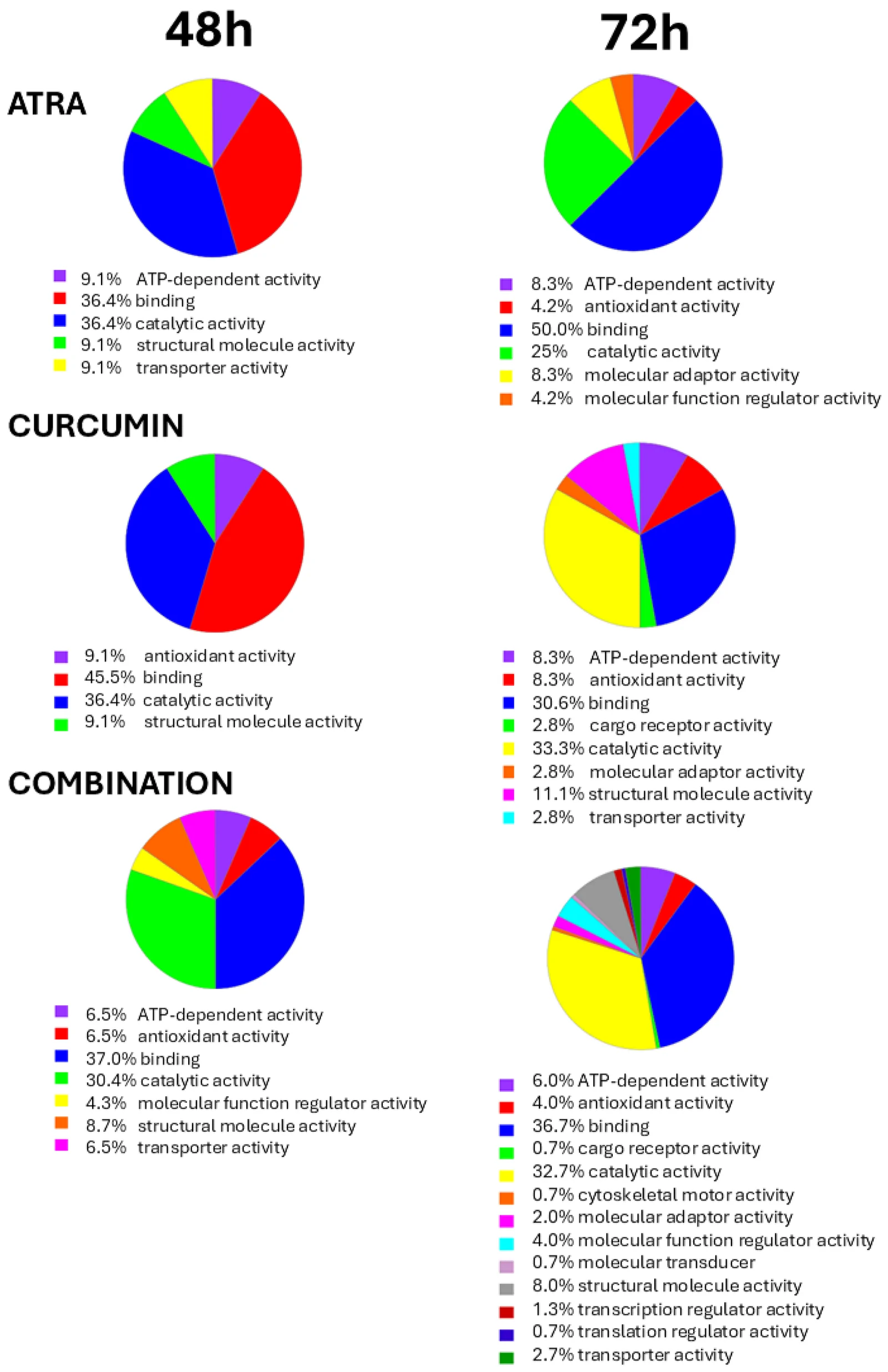
Molecular functions of proteins modified by ATRA and curcumin alone and in combination at 48 h and 72 h. Abbreviations: ATRA: All-trans Retinoic Acid.

Gene ontology analyses were performed to classify the molecular functions and pathway associations of proteins that differed between groups. Figure 7 presents the molecular functions. According to these analyses, binding and catalytic activity accounted for more than 25% of all drug groups and time intervals. ATP-dependent activity was also a common molecular function, occurring between 6.0% and 9.1% across all drug groups and time intervals, except in the curcumin-treated group at 48 h. In both time intervals, the distributions of molecular functions in the combination group resembled the union of those in single-drug applications. At 48 h, structural molecular activity occurred in all drug groups at rates between 8.7% and 9.1%, while transporter activity occurred in the ATRA and combination groups. Notably, curcumin’s effect at 48 h included antioxidant activity, but not ATP-dependent or transport activities.

Supplementary Figures S1 and S2 present the pathway associations of differentially expressed proteins at 48 h and 72 h, respectively. At 48 h, 50% of the proteins altered by ATRA administration were associated with serine-glycine biosynthesis, and 25% each with pyridoxal-5-phosphate and vitamin B6 metabolism. Curcumin-induced changes clustered under five groups with equal percentages: TCA cycle, ATP synthesis, apoptosis, gonadotropin-releasing hormone (GnRH) receptor pathway, and integrin signaling. Pathway associations in the combination group were much more widely distributed. Among these, the GnRH and Parkinson’s disease pathways were most prominent, each accounting for 14.3%, while the remaining pathways accounted for 7.1% each. These were associated with purine, pyrimidine, pyruvate, asparagine, and aspartate metabolism; Wnt, cadherin, and integrin signaling; and the ubiquitin–proteasome, cell cycle, and apoptosis pathways. At 72 h, proteins changed in the ATRA group were distributed equally across six physiopathological processes or pathways: serine-glycine biosynthesis, DNA replication, Parkinson’s disease, VEGF signaling pathway, and angiogenesis.

Curcumin-induced changes at 72 h were distributed equally across 11 different pathways. These included de novo pyrimidine ribonucleotide and deoxyribonucleotide synthesis, DNA replication, glycolysis, the pentose phosphate pathway, fructose and galactose metabolism, integrin signaling, cholecystokinin receptor (CCKR), and chemokine- and cytokine-mediated inflammation. At the same interval, combination therapy resulted in a broad distribution of pathways, with none alone accounting for more than 5.2% of the total detected pathways. The most prominent of these were the integrin signaling pathway (5.2%), Parkinson’s disease (5.2%), Huntington’s disease (4.1%), chemokine- and cytokine-mediated inflammation (4.1%), and ubiquitin–proteasome pathway (3.1%). For the 51 identified pathways, the percentage values within the total group were mostly around 1%, with none exceeding 2.1%. These findings suggest that ATRA and the combination group share enrichment in the Parkinson’s disease and Huntington’s disease pathways, both of which are linked to cellular neurodegeneration. Furthermore, the curcumin and combination groups showed overlap in the CCKR, GnRH, integrin, and inflammation pathways. Given the emergence of proteins associated with neurodegeneration and inflammation, as well as the neuroectodermal origin of GBM cells and their similarities to nerve cells, the results can be interpreted as the onset of intense cellular stress.

### 2.6. Individual Proteins Showing Differential Expression Across Treatment Groups

Supplementary Table S2 lists the complete set of proteins showing statistically significant changes across all groups. Because the number of these proteins was too high, two criteria were applied to select proteins likely to have the most discernible roles in cellular responses to drugs and to be considered in the discussion. First, among proteins showing statistically significant changes, those with |Log2FC| ≥ 3 in at least one treatment group were identified. Second, among proteins showing statistically significant changes, those showing differences across all treatment groups and with |Log2FC| ≥ 1.5 were selected. The reason for this choice was to simultaneously reveal proteins with profound expression changes and those with relatively few changes, yet observable across all treatment groups and with distinct trends. This was aimed at obtaining a more comprehensive understanding of the synergistic interactions among agents that antagonize their effects at low doses. A |Log2FC| value of 1.5 corresponds to approximately a 2.83-fold increase or decrease in protein levels and is considered a medium-to-high level of change in proteomic studies. In this study, to prevent small differences from becoming statistically significant due to the large-sample-size trap, proteins were selected with a strict false discovery rate (FDR) value of 0.01 using the Benjamini–Hochberg correction. Furthermore, the −log10(*p*) values of the proteins selected based on a 1.5 |Log2FC| value were 1.979–5.898. This means that their *p*-values for significance ranged approximately from 0.01 to 1.26 × 10^−6^, indicating statistically very large differences. Therefore, the selected proteins appear highly likely to represent true biological responses. Proteins having |Log2FC| values ≥ 3 indicate increases or decreases of ninefold or more, and at an FDR level of 0.01, these can be considered components of strong cellular responses to drug effects. Table 2 lists all individual proteins identified using the two criteria described above.

Based on these criteria, 11 proteins emerged as the most prominent. Overall, more proteins exhibited marked changes in the ATRA group than in the curcumin group, consistent with ATRA’s stronger inhibitory effects on cell growth and migration. These included significant reductions in tenascin, Transforming Growth Factor Beta Induced (TGFBI), Phosphoglycerate Dehydrogenase (PHGDH), Nucleobindin 2 (NUCB2), and Sequestosome 1 (SQSTM1), among which tenascin was downregulated only in the ATRA group, TGFBI and PHGDH in the ATRA and combination groups, and NUCB2 and SQSTM1 in all groups. Curcumin lessened ATRA-induced decreases in TGFBI and PHGDH and completely abolished this effect on tenascin, while augmenting ATRA’s effect of lowering SQSTM1 levels. Sad1 and UNC84 Domain Containing 2 (SUN2) increased in both the ATRA and curcumin groups, a change that peculiarly disappeared with combined treatment. The expression of Uveal Autoantigen With Coiled-Coil Domains And Ankyrin Repeats (UACA), Transmembrane 9 Superfamily Member 2 (TM9SF2), and Ribosomal Protein L37a (RPL37A) proteins was enhanced in all groups. Among single treatments, UACA and TM9SF2 showed the largest increases in ATRA and RPL37A in the curcumin group, respectively. Curcumin reduced ATRA’s efficacy in upregulating UACA, while potentiating ATRA-induced increases in TM9SF2 and RPL37A protein levels. Two proteins manifested peculiar expression patterns. In single-agent groups, Hexokinase-2 was upregulated only in the curcumin group, but this upregulation was further increased in combination, despite ATRA’s inability to elevate its expression. Lastly, although Heme Oxygenase 1 (HMOX1) did not change with single agents, it was strongly reduced with the ATRA–curcumin combination.

### 2.7. Protein–Protein Interactions (PPIs)

**Figure 8:**
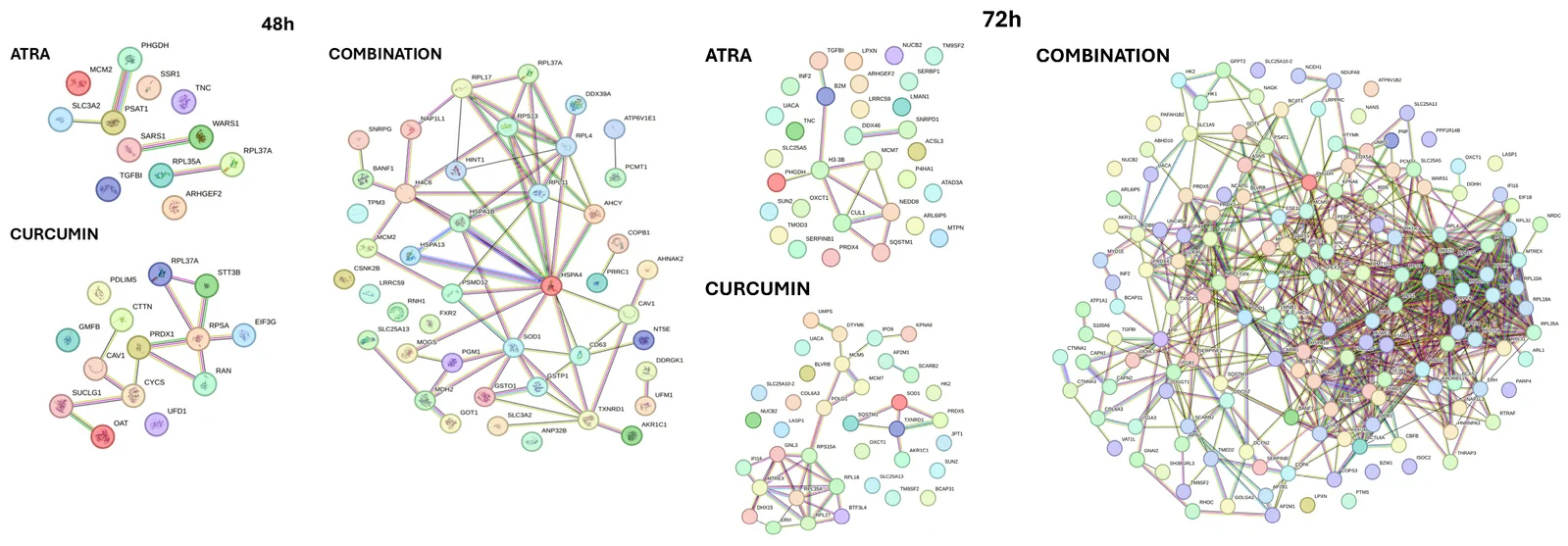
Protein-protein interactions (PPIs)defined for the ATRA, curcumin, and combination groups at 48 h and 72 h. Abbreviations: ATRA: All-trans Retinoic Acid.

Figure 8 demonstrates PPIs emerging with drug treatments during single and combined applications. The STRING database analysis of known and predicted PPIs revealed distinct interaction clusters for different treatments. At 48 h in the ATRA group, Phosphoserine Aminotransferase 1 (PSAT1) and PHGDH proteins were linked, reflecting changes in serine biosynthesis and metabolic regulation. At the same interval in the ATRA group, Peroxiredoxin-1 (PRDX1) and Ribosomal protein SA (RPSA) were identified as associated, with both being involved in redox regulation and ribosomal function. In the combination group at 48 h, Heat shock 70kDa protein 4 (HSPA4) emerged as a central protein, interacting with many proteins (HSPA1B, HSPA13, PSMD12, GSTP1, and SOD1) associated with stress response, protein folding, and metabolism. The network structures obtained for 72 h showed more extensive clusters. In the ATRA group, histone H3.3 (H3-3B) and PHGDH proteins were identified as central hubs. Cullin 1 (CUL1), Neural precursor cell expressed developmentally downregulated protein 8 (NEDD8), and SQSTM1 together formed a tight network associated with protein degradation and ubiquitin–proteasome proteins, which also involved DEAD-box helicase 46 (DDX46) and Small nuclear ribonucleoprotein Sm D1 (SNRPD1). In the curcumin group at 72 h, ribosomal proteins (RPL35A, RPL27, RPL18, RPS15A) and RNA processing factors (DHX15, ERH, BTF3L4, MTREX) formed a tight network demonstrating coordinated alterations of ribonucleoprotein complexes. Another cluster included proteins involved in DNA replication and repair (MCM5, MCM7, POLD1, DTYMK), suggesting that curcumin could also modify DNA synthesis and integrity. Additionally, a redox network comprising Superoxide dismutase 1 (SOD1), Thioredoxin Reductase 1 (TXNRD1), Peroxiredoxin-5 (PRDX5), and Aldo-Keto Reductase Family 1 Member C1 (AKR1C1) proteins emerged as a distinct hub associated with antioxidant responses. The combination group showed the most extensive and densely linked clusters associated with regulators of ribosomes, DNA replication, the cell cycle, and redox balance, which exhibited a coordinated pattern at 48 h but shifted to an inextricable state at 72 h.

## 3. Discussion

### 3.1. ATRA and Curcumin Effects on Cell Proliferation and Migration

**Figure 9:**
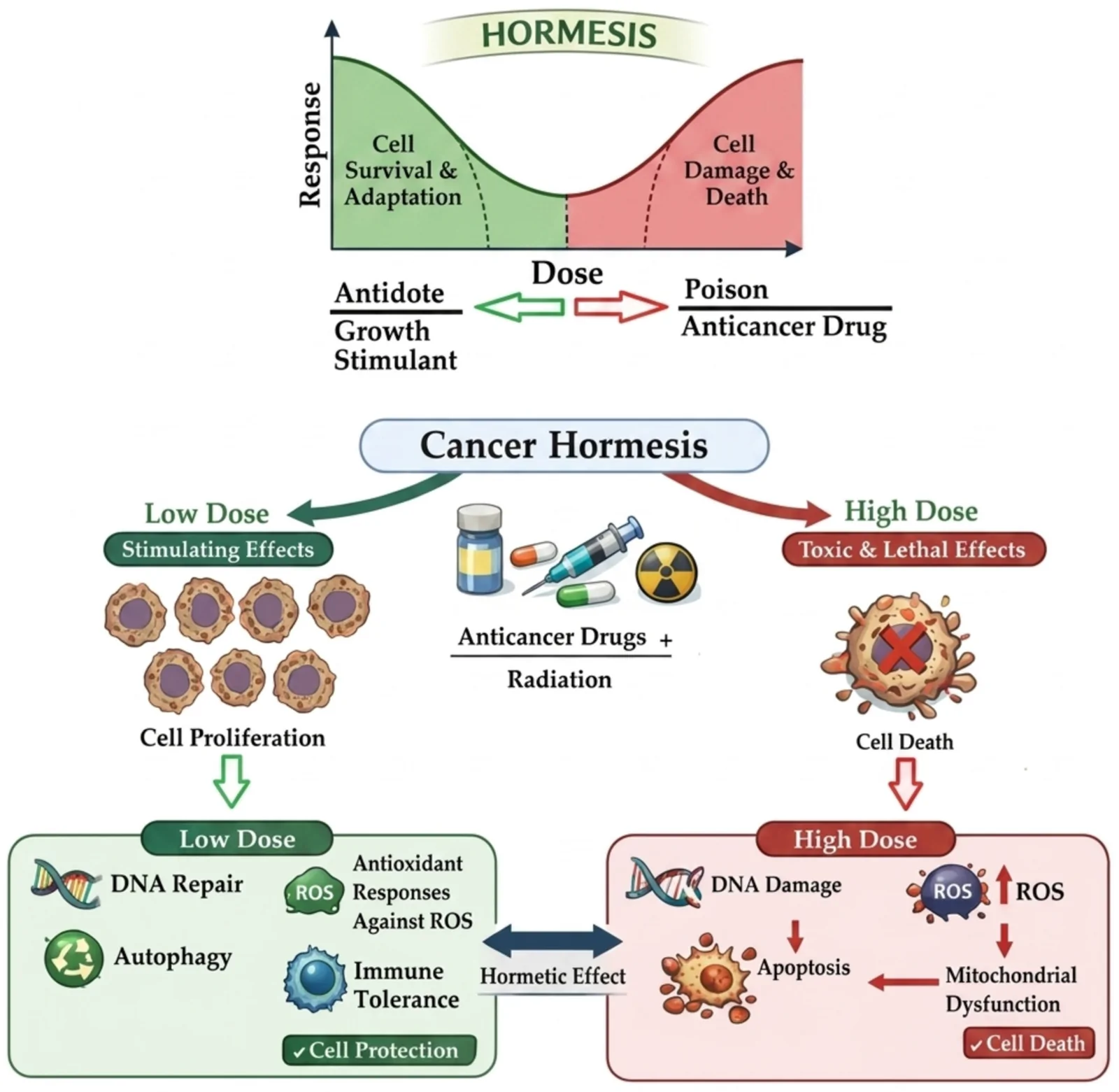
The hormesis concept and its cellular mechanisms in general and cancer medicine. (Created in BioRender. OZPINAR, A. (2026) https://BioRender.com/ij001fu, accessed on 14 August 2026).

A notable outcome of this study is that low doses of ATRA and curcumin stimulated cell growth and suppressed the antiproliferative effects of the other, both effects consistent with hormesis. Hormesis provides an explanation for mithridatism, known from the practices of King Mithridates (135–63 BC), which involves conferring immunity to poisons by consuming them at low but gradually increasing doses, and the “dose determines the toxicity” concept proposed by Paracelsus (1493–1541) [24]. In contemporary medicine, hormesis describes how substances at low doses, even with potential toxicity, can act as antidotes at the systemic level or as tumor promoters by increasing the adaptation, survival, and proliferation of healthy or malignant cells, while acting as a systemic poison or an anticancer drug by intensifying cell damage at higher doses (Figure 9). At the cellular level, the effects of hormesis manifest as increased DNA repair and antioxidant defense against ROS, activation of autophagy to break down damaged organelles and the use of emerging biomaterials as building blocks, and even low-dose immune responses promoting cancer growth [25,26,27,28]. At high drug doses, hormesis pathways are lost, DNA damage and ROS reach intolerable levels, and DNA and mitochondrial injuries culminate in apoptosis through mechanisms that may begin as distinct pathways but later reinforce each other [29,30]. Notably, low-dose ROS act as second messengers in both benign and malignant cells, promoting differentiation in the former and proliferation in the latter [31].

Another important study result is that ATRA and curcumin at their highest doses synergistically inhibited cell migration, which is not contradictory to their effects at low doses. Because the migration test did not capture extracellular matrix features, the observed antimigratory effects are more plausibly attributable to cytoskeleton-modifying mechanisms shared by both agents than to matrix-degrading enzymes. Indeed, retinoic acid dose-dependently regulates cytoskeletal organization and epicardial cell migration during cardiac embryogenesis, and its high levels are associated with cleft palate development by disrupting cytoskeletal architecture and palatal mesenchymal cell migration through mechanisms involving Rho GTPase Cdc42 and Wnt5A [32,33].

In parallel, curcumin alters the epileptic mouse brain proteome primarily regarding cytoskeletal proteins, and it inhibits U87 GBM and SGC-7901 gastric cancer cell migration by disrupting focal adhesion complexes in the former and cytoskeletal proteins, Shh, and Wnt signaling in the latter, respectively [34,35]. The mouse brain findings are notable because curcumin is generally considered unable to cross the brain barrier despite its traditional East Asian medicinal use for epilepsy [35].

### 3.2. Proteins Mediating Hormesis Versus Anticancer Effects of ATRA and Curcumin

At all dosages, ATRA stimulated cell proliferation at 24 h, slightly affected cell growth at 48 h, and dose-dependently inhibited U87 GBM cell density at 72 h. At 72 h, ATRA at 75 µM induced signs of early and late apoptosis in 53.3% and 13.5% of cells, respectively. These findings suggest that ATRA initially acted hormetically on cell metabolism and growth, while a relatively low percentage of late apoptosis at 72 h indicates anabolic effects that probably persist beyond 48 h but are not reflected in cell counts. In parallel, ATRA alone mostly affected anabolic serine-glycine biosynthesis, pyridoxal-5-phosphate, and vitamin B6 metabolism, with the latter two effects vanishing at 72 h, accompanied by the emergence of pathways associated with DNA replication and Parkinson’s disease. Reduction of the versatility of anabolic cascades and the emergence of DNA replication and Parkinson’s disease pathways may suggest enhanced DNA injury and cell degeneration onset, as specific proteins involved in DNA replication are also involved in DNA repair, and molecular steps of malignant and neuronal cell death have similar features [36,37,38]. The relatively less-known tumorigenic effects of retinoic acid have been observed in prostate cancer cell cultures and in trials conducted for liver cancer and even for acute myeloid leukemia [39]. These actions may involve ROS, as retinoic acid-induced neural differentiation is mediated by ROS signals with a tight concentration threshold determining second messenger functions or toxicity [12]. Unlike many other cancers, glial tumors express higher levels of Cellular Retinol-Binding Protein 1 (CRBP1) and Aldehyde dehydrogenase 1 family member A1 (ALDH1A1), which expedite cellular retinoid uptake and mediate active retinol metabolite synthesis, respectively [40]. Despite these features, CRBP1 and ALDH1A1 expression levels correlate with worse survival rates in patients with glioma, contrary to the expected antineoplastic actions of retinoids [40]. Nonetheless, ATRA may still play a role in GBM treatment, as evidenced by sparse clinical trials and other molecular influences, which will be detailed below during the discussion of individual proteins associated with drug actions.

Curcumin at low doses induced greater hormesis, reflected by higher growth-stimulatory effects as a single agent and more potent inhibitory effects on the antineoplasticity of its partner drug in combination. In addition, curcumin at its highest dose was less potent than ATRA in reducing cell growth and migration. All these effects can be ascribed to curcumin’s modification of distinct protein sets. At 48 h, curcumin differed from the ATRA and combination groups by the emergence of proteins involved in antioxidant responses and the absence of changes in proteins with structural molecular activity, which reflects the protective effects of curcumin against ROS and its inefficiency in markedly modifying proteins that act as cellular building blocks. Further, curcumin-induced proteins at 48 h were associated with the TCA cycle, ATP synthesis, GnRH receptor, and apoptosis pathways, reflecting the activation of energetic and presumably anti-apoptotic cascades, which parallel the most profound stimulation of cell growth at this time point. Again at 48 h, curcumin acted more efficiently than ATRA to enhance the RPL37A protein involved in ribosomal functions, and at 72 h, it altered the levels of proteins with links to glycolysis, pentose phosphate, fructose, galactose, nucleotide synthesis, and DNA replication pathways and increased Hexokinase-2 enzyme levels not modified by ATRA alone. All these features indicate that curcumin’s stimulation of cell metabolism may persist beyond 48 h, even in the absence of changes in cell density. At the same interval, curcumin-induced proteins are associated with inflammatory pathways, which may reflect the onset of cellular stress, as proteins mediating cell death are linked to inflammasome activation. Additionally, the emergence of proteins involved in nucleotide synthesis and DNA replication may result from DNA injury induced by the highest curcumin dose.

ATRA and, to a lesser extent, curcumin enhanced UACA and SUN2 proteins, effects that were either reduced or disappeared when combined, features that may reflect a collapse of hormesis and a shift to synergistic anticancer effects. Curcumin attenuated or completely reversed ATRA-mediated decreases in the tumorigenic TGFBI, PHGDH, and tenascin proteins, which likely resulted from curcumin’s induction of greater hormesis and antagonism of ATRA effects, which may persist despite the appearance of synergistic antineoplasticity. Curcumin potentiated ATRA-induced increases in RPL37A and TM9SF2 proteins and a decrease in SQSTM1 levels, with the first two effects more likely related to ATRA and curcumin hormesis that continued despite the emergence of anticancer synergism, and the last effect contributing to their antineoplasticity alone and in synergy when combined. Hexokinase-2 protein was increased with curcumin and more so with combined treatment, despite not being induced by ATRA, which suggests that ATRA likely induces a permissive state for curcumin’s specific molecular actions. Lastly, the HMOX1 protein was significantly depleted in the combination, despite no change with either agent alone, suggesting a disrupted cellular signaling network.

UACA (Uveal autoantigen with coiled-coil domains and ankyrin repeats) was isolated from embryonal carcinoma cells as a pro-apoptotic protein [41]. However, later organismal studies demonstrated effects opposite to those observed in cell culture. Most importantly, UACA binds and neutralizes the pro-apoptotic anticancer protein PAR-4 in the blood, which is released from healthy cells in proportion to the level of wild-type p53, a major tumor suppressor [42]. In parallel, UACA levels are upregulated in human hepatocellular carcinoma tissues compared with benign liver parenchyma, and its knockdown in liver cancer cells results in decreased cell proliferation and migration and enhanced senescence [43]. Because ATRA and curcumin increased UACA levels individually but to a lesser extent in combination, UACA may contribute to hormesis and begin to decline under intense cellular stress. However, firm conclusions cannot be made at this time, given its initially demonstrated pro-apoptotic functions, and further experiments are needed to clarify its role in GBM biology. While SUN2 was significantly upregulated by ATRA and curcumin alone, it was completely undetectable in combination therapy. SUN2 is an inner nuclear membrane protein that connects the nucleoskeleton and cytoskeleton and plays dual roles in cancer, as shown for UACA. SUN2 acts as a tumor suppressor in breast cancer by repressing a Ca^2+^/calcineurin-responsive transcription factor, Nuclear Factor of Activated T-cells, Cytoplasmic 4 (NFATC4) [44]. Yet, it is also upregulated in an aggressive human brain neoplasm and in medulloblastoma and is correlated with significantly shorter survival [45]. SUN2 may be a component of hormesis induced by both drugs when applied alone. However, it may also be a component of ATRA- and curcumin-mediated anticancer effects, which need to be investigated. Lastly, the disappearance of SUN2 observed in the combination group may result from severe proteolysis, which may have accompanied either the loss of hormesis induced by ATRA and curcumin or the increased anticancer effects of their combination.

The greatest modification in fold change, with robust statistical significance, was observed in TGFBI reduced by ATRA. TGFBI is a protein with an RGD motif that serves as a ligand recognition sequence for integrin proteins and cell adhesion. The existing literature on TGFBI’s role in GBM biology commonly suggests pro-tumorigenic functions. TGFBI expression is significantly higher in glial tumor tissues than in normal brain parenchyma, especially in the mesenchymal subtype, which is associated with a poor prognosis, and elevated levels correlate with shorter patient survival rates [46]. Furthermore, hypoxia-induced maintenance of an aggressive stem cell population in GBM involves HIF1α-mediated upregulation of TGFBI, which stimulates the AKT/c-myc signaling pathway [47]. Lastly, TGFBI induces epithelial–mesenchymal transition, migration, and invasion of GBM cells [48]. Overall, strong TGFBI depletion after high-dose ATRA may underlie its inhibition of cell growth and migration, while the weaker depletion observed with the combined treatment may indicate partially persistent hormetic activity of curcumin.

Another protein strongly depleted by ATRA is tenascin, an extracellular matrix glycoprotein expressed at high levels during embryogenesis, wound healing, and in tumor stromal tissues. The extensive literature on tenascin in GBM biology consistently indicates that it has tumor-stimulating actions. These include results showing increased intratumoral tenascin expression in correlation with higher pathological grades of glial tumors, expression in specific GBM stem cell subpopulations with a high self-renewal potency, and promotion of vascularization, cell growth, invasive features, and an immunosuppressive microenvironment within GBM tissues [49,50,51]. Hence, ATRA-induced profound depletion of tenascin likely contributed to the antiproliferative and anti-invasive effects, and the complete reversal of ATRA-induced changes in combination likely results from sustained hormesis pathways triggered by curcumin, despite the emergence of a synergistic anticancer effect. ATRA also downregulated the PHGDH protein, an enzyme that catalyzes the conversion of 3-phosphoglycerate to 3-phosphohydroxypyruvate, a critical step in the synthesis of serine, nucleotides, and other metabolites crucial for cell proliferation. This expression change is consistent with the STRING analysis, which shows that the serine synthesis pathway stands out under ATRA treatment. Scientific studies have also demonstrated significant tumor-stimulating effects of PHGDH in glial tumors, with consensus. These findings revealed that PHGDH promotes temozolomide resistance in GBM cells, that HIF-1α induces PHGDH expression together with proteins that boost glycolysis, and that PHGDH stimulates stem cell renewal, DNA damage responses, and the myc oncoprotein in GBM cells [52,53,54]. Again, alleviation of the ATRA-mediated reduction in PHGDH levels with combined treatment may reflect a persisting hormesis metabolism induced by curcumin, masked in the proliferation and migration data, since it parallels curcumin’s established roles in stimulating the DNA damage responses.

Ribosomal 60S subunit protein RPL37A and TM9SF2 were enhanced by ATRA and to a greater extent by curcumin, effects that were further increased in combination. All these effects may result from hormesis induced by both drugs, with the perseverance of certain anabolic components despite the onset of synergistic anticancer effects. Indeed, higher RPL37A expression was correlated with higher glial tumor grades and worse survival rates in patients with GBM, which may be attributed to higher protein synthesis and an elevated anabolic state in tumor cells [55,56]. Findings regarding the role of the TM9F2 protein in cancer biology are similar to those relevant to RPL37A. TM9SF2 is involved in endosomal trafficking, small-molecule transport, and Golgi integrity, acts as an oncoprotein in pancreatic and colorectal cancers, and hypothetically functions as an ion channel [57,58]. Supporting the assumption that TM9SF2 contributed to the hormesis and tumorigenic actions elicited by ATRA and curcumin, with its levels synergistically increased in combination, a recent study revealed that higher TM9SF2 levels were correlated with shorter survival in pediatric patients with glial tumors [59].

ATRA and curcumin reduced the SQSTM1(Sequestosome-1) protein levels individually, with a stronger reduction in combination, suggesting contributions to their anticancer effects and antineoplastic synergism. SQSTM1 protein regulates proteostasis and autophagy by binding ubiquitin and bridging ubiquitinated proteins to autophagosomes for degradation. Autophagy has opposing effects on cell survival, which could cause cell death if it is extensive, but it often provides cellular resilience against stress conditions through the degradation of damaged proteins and organelles, which provides several benefits for cells by alleviating the harm that could arise due to dysfunctional proteins and organelles, providing biomaterial for the salvage synthesis of essential macromolecules, and by the activation of the anti-apoptotic and pro-tumorigenic transcription factor NF-κB [60]. In parallel to the beneficial roles of autophagy for cancer cell resilience, SQSTM1 expression increases in higher-grade gliomas, induces temozolomide resistance in GBM cells by activating selective autophagy, and promotes GBM cell growth and migration, with the last effect appearing to be related more to its possible anabolic functions, which are less reported in the current literature [61,62,63].

Among the proteins with the highest fold-change in expression, HXK2 was the only protein not influenced by ATRA but increased by curcumin and to a greater extent by the combination. HXK2 is an outer mitochondrial membrane enzyme that catalyzes the first step in glycolysis, on which malignant cells mostly depend, and it exists at high levels in striated muscle and rapidly growing malignant cells. HXK2 is expressed more in glioma than in benign brain tissues, with increased quantities correlating with a worse prognosis, and its silencing reduced cell growth, migration, tumorigenicity, and vasculogenic mimicry in GBM models [64,65]. All these features once more indicate at least partially continuing hormesis induced by curcumin, despite the manifest anticancer synergism, in which ATRA may have acted as a permissive factor.

The last protein deserving discussion is HMOX1, which was not influenced by either agent alone but was remarkably reduced with combined treatment. HMOX1 (heme oxygenase 1) protein is an enzyme that breaks down heme and is essential for cellular resilience against ROS, which is also induced by other detrimental factors such as hypoxia and heavy metals, thereby exerting cytoprotective functions. The available research on HMOX1 involvement in GBM biology yielded contradictory outcomes, as was the case with several other proteins reviewed in this study. However, a tumorigenic function was attributed to HMOX1 in most relevant articles. Similar to some other proteins discussed above, elevated HMOX1 expression relates to higher-grade glial tumors and worse prognosis, induces mesenchymal dedifferentiation, and promotes driver gene mutations and other oncogenic transformations, as well as ferroptosis evasion with subsequent radiation resistance, as observed in pathological, clinical and experimental studies [66,67,68]. Hence, the prominent decrease in HMOX1 levels may be a primary factor underlying the anticancer synergism of ATRA and curcumin, whereby cells lose the ability to coordinate and synchronize stress defense mechanisms. This proposal is debated in more detail in the succeeding section, which describes the putative reason for the inordinate diversification of protein pathways in the combination group.

### 3.3. Excess Protein Diversification During Shift from Hormesis to Anticancer Synergism

In this study, molecular function, pathway, andPPI analyses demonstrated a shared feature. During high-dose treatments with single agents, changes in protein levels and interactions at 48 h were limited and exhibited a focused pattern. By 72 h, this pattern relaxed slightly as new proteins and interactions emerged. In contrast, combination therapy activated a broader range of pathways and interactions at 48 h, with changes being significantly amplified at 72 h, resulting in a robust diversity comprising elements with minute percentages. Two possible explanations may account for these observations. First, GBM cells initially diversify resistance pathways to protect against the distinct actions of ATRA and curcumin, and then further augment these to enhance cellular defenses. Second, excessive proteomic diversity may indicate intracellular chaos rather than adaptation, as it coincides with the synergistic inhibition of proliferation and migration. Thus, extensive proteomic changes may result from a loss of coordinated cellular responses to stressors. This view is supported by the emergence of proteins associated with neurodegenerative and inflammatory pathways, especially at 72 h with combined treatment, suggesting signaling disorganization rather than protective mechanisms. The hypothesis that an inordinate proteomic diversification indicates stochastic changes rather than synchronized cellular reactions and a shift from hormesis toward antineoplastic synergism shall be tested in further experiments. If this proposal is relevant, new treatment strategies may be developed by determining the drug exposure-associated ‘point of no return’ proteomic thresholds to predict clinical responses.

## 4. Materials and Methods

**Figure 10:**
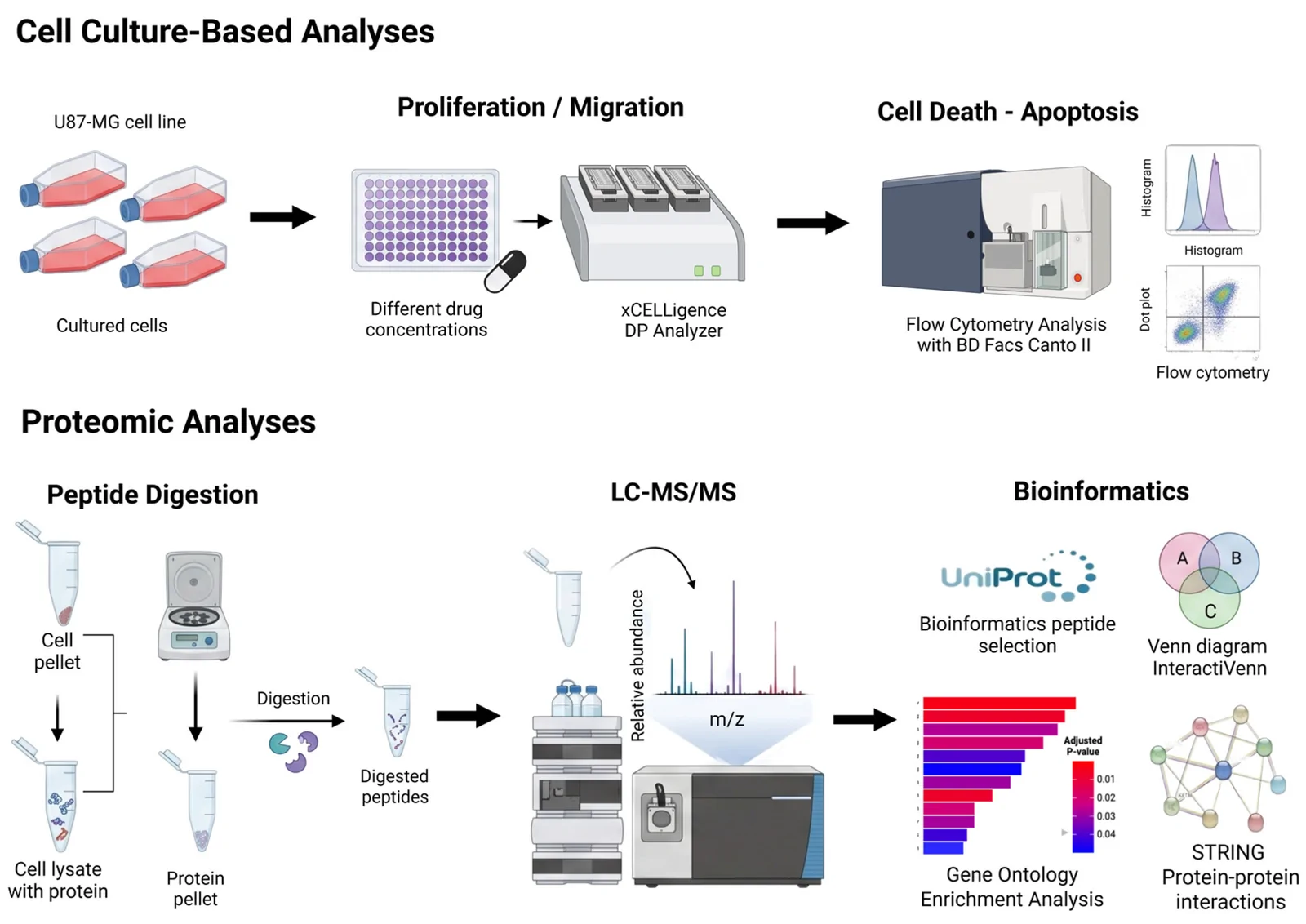
Experimental workflow including cell culture and proteomic analysis steps. (Created in BioRender. OZPINAR, A. (2026) https://BioRender.com/ij001fu, accessed on 14 August 2026).

Figure 10 demonstrates the experimental workflow utilized in this study.

### 4.1. Chemicals and Cell Culture Conditions

The American Type Culture Collection (ATCC) (Manassas, VA, USA) supplied the human U87 GBM cell line (MG subtype, ATCC HTB-14). All-trans retinoic acid (Merck, 554720, Rahway, NJ, USA) and curcumin (Merck, 239802) were stored and dissolved in DMSO (Sigma, D2650, St. Louis, MO, USA) according to the manufacturer’s instructions. ATRA and curcumin stock solution concentrations were 31.06 mM and 13.15 mM, respectively, which were diluted to the desired concentrations during experiments. Gibco DMEM (Waltham, MA, USA, Dulbecco’s Modified Eagle’s Medium, High Glucose with normal L-Glutamine, 500 mL) was the basic medium and was aliquoted into 45 mL volumes. For the complete medium, 10% fetal bovine serum (Sigma, F7524) (5 mL) and 1% Penicillin–Streptomycin (Thermo, 15140122, Waltham, MA, USA) (0.5 mL) were used. The flasks were kept in an incubator with a humidified atmosphere of 5% CO_2_ at 37 °C. After the cells covered 70–80% of the plate surface, the cells were harvested with trypsin-EDTA (Thermo, 25200056). Trypan blue (GLPBIO, GF00376) and a BIORAD Cell Titer were used for viable cell identification and counting, respectively. The viable cells (1 × 10^6^) were transferred into flasks containing complete medium. In all groups, the final DMSO concentration was adjusted to 0.2% to ensure equal solvent conditions across all wells.

### 4.2. xCELLigence Cell Proliferation Assay

The xCELLigence RTCA (Real-Time Cell Analyzer) DP (dual-purpose) device is an advanced instrument that enables real-time imaging of cellular density and migration through impedance measurements by microelectronic sensors. During experiments, the device was kept in a standard 37 °C, 5% CO_2_ cell culture incubator to ensure that the cells were kept under appropriate conditions. A total of 50 µL of medium was added to the E-plates for background measurement. Then, U87 GBM cells were prepared in 100 µL of medium at 5000 cells/well and seeded into the wells. E-plates were incubated at room temperature (20–25 °C) for 30 min, then placed in the device for incubation. After 24 h of incubation for the cells to attach to the wells and enter the logarithmic growth phase, the cells were treated with each drug alone and in combination at the desired concentrations (ATRA: 25 µM, 50 µM, and 75 µM; curcumin: 5 µM, 10 µM, 20 µM). The E-plates were then inserted into the xCELLigence device to monitor cell growth in real time. After 72 h of drug administration, the experiment was completed, and the data were analyzed using RTCA 2.1.0 software. All experiments were performed in triplicate.

### 4.3. Calculation of the Combination Index

CI values were calculated using CompuSyn software (ComboSyn Inc., New York, NY, USA) to determine whether drug interactions were synergistic, additive, or antagonistic. Because the low doses of ATRA and curcumin administered caused partial cell proliferation at 48 h and CompuSyn software could not account for proliferative stimulations in the analysis, CI analyses could only be performed at 72 h. The FCA values represent the percentage of cells inhibited by drug treatment relative to the untreated control group. For instance, an FCA value of 0.609 represents a 60.9% inhibition of growth. The CI value indicates the interaction between the two drugs: a CI below 1 suggests a synergistic effect, values between 1 and 1.2 indicate an additive effect, and values above 1.2 indicate an antagonistic interaction.

### 4.4. xCELLigence Cell Migration Assay

The effects of ATRA (75 µM), curcumin (20 µM), and their combination on cell migration were monitored using the xCELLigence DP system with CIM-Plate 16, following the manufacturer’s instructions. The cells were serum-starved in a medium containing 1% FBS for 12 h before the experiment. For the migration assay, 30,000 cells suspended in a medium with 1% FBS were seeded into each well of the upper chamber (UC). The lower chamber (LC) was filled with a medium containing 10% FBS as a chemoattractant. The plate was incubated in the hood for 30 min. After this incubation, ATRA, curcumin, and their combination were added to the designated wells (*n* = 3 per condition). Impedance values were recorded over a 72-h period using the xCELLigence system, and the resulting NCI values were analyzed using GraphPad Prism software (version 8.0).

### 4.5. Flow Cytometric Analysis

The apoptotic effects were evaluated using the FITC Annexin V Apoptosis Detection Kit I (BD Biosciences, Cat. No.: 556547, San Diego, CA, USA) and analyzed with a BD FACS Canto II flow cytometer. U87 GBM cells were seeded at 1 × 10^5^ cells per well in 6-well plates, incubated for 24 h, and then treated with ATRA (75 μM) or curcumin (20 μM) as single agents or in combination. The control groups received only DMSO at the same concentration. At 48 and 72 h, cells were harvested to analyze apoptosis rates. First, the culture medium in each well was removed, and the cells were washed with PBS. The medium and washing solution were transferred to tubes. Adherent cells were detached and then added to the respective tubes. Cell suspensions were centrifuged at 400× *g* for 5 min, and the supernatant was discarded. The resulting cell pellets were washed with cold PBS and resuspended in a 1X binding buffer following the manufacturer’s instructions. For apoptosis staining, Annexin V-FITC and PI were added to each sample, following the manufacturer’s recommended concentrations. The samples were gently vortexed and incubated in the dark at room temperature for 15 min. Following incubation, the samples were analyzed using a BD FACS Canto II flow cytometer. Data were processed and analyzed using FlowJo software (v10.10, BD Biosciences). All experiments were performed in triplicate.

### 4.6. Statistical Analysis of Non-Proteomic Data

Cell proliferation data were analyzed using two-way ANOVA followed by Tukey’s multiple comparisons test in GraphPad Prism (version 9.0, GraphPad Software, San Diego, CA, USA). Migration data were evaluated by two-way ANOVA with Sidak’s multiple comparisons test to determine group differences over time. A significance threshold of *p* < 0.05 was considered statistically significant.

### 4.7. Sample Preparation for Proteomic Analysis

Cells treated with ATRA, curcumin, and combination drugs at specified doses, as well as control group cells, were collected using a scraper after 48 and 72 h of incubation, and the resulting cell pellets were stored at −80 °C for proteomic analysis. All proteomic analyses were designed as triplicates. In-solution digestion was used to prepare the protein samples, and mass spectrometry (MS) analyses were performed as described below. Frozen cell pellets were lysed with 8 M urea in 50 mM ammonium bicarbonate buffer (Sigma-Aldrich, St. Louis, MO, USA) containing a cOmplete™ EDTA-free protease inhibitor tablet (Roche Diagnostics GmbH, Mannheim, Germany) and a PhosSTOP™ phosphatase inhibitor tablet (Roche Diagnostics GmbH, Mannheim, Germany). Subsequently, the samples were reduced with dithiothreitol (DTT; Sigma-Aldrich, St. Louis, MO, USA) and alkylated with chloroacetamide (Sigma-Aldrich, St. Louis, MO, USA). The samples were diluted with 50 mM ammonium bicarbonate (Sigma-Aldrich, St. Louis, MO, USA) and incubated overnight with Sequencing Grade Modified Trypsin (Promega Corporation, Madison, WI, USA) at 37 °C (16 h). Following the acidification step, trypsin-digested peptides were desalted using a C18 Sep-Pak system (Waters Corporation, Milford, MA, USA). The resulting peptides were resuspended in a solution containing 5% formic acid (Thermo Fisher Scientific, Waltham, MA, USA) and 5% acetonitrile (Thermo Fisher Scientific, Waltham, MA, USA) prior to nano-LC-MS/MS analysis.

### 4.8. Mass Spectrometry

Peptides generated by LC-MS/MS were analyzed using an online C18 nanoflow reversed-phase HPLC system (Dionex Ultimate 3000 RSLC nano, Thermo Fisher Scientific, Bremen, Germany) coupled to an Orbitrap mass spectrometer (Q Exactive Orbitrap, Thermo Fisher Scientific, Bremen, Germany). Peptides were loaded onto a laboratory-packed C18 column (ReproSil-Pur 120 C18, 1.9 μm resin, Dr. Maisch GmbH, Ammerbuch-Entringen, Germany) with an inner diameter of 75 μm × 35 cm in length and separated using increasing acetonitrile gradients in 0.1% formic acid for a total run time of 90 min (4–25%, 25–40%, and 40–95% acetonitrile; flow rate 300 nL/min). Scans were initiated with the MS1 spectrum (analysis parameters: resolution 70,000; mass range 400–1500 *m*/*z*; automatic gain control (AGC) objective 3 × 10^6^; maximum injection time 60 ms). In each cycle, the 15 most intense ions were selected, fragmented, and analyzed in the Orbitrap detector in data-dependent acquisition (DDA) mode. In the MS2 analysis, higher-energy collisional dissociation (HCD) was applied (resolution 17,500; AGC target 5 × 10^4^; normalized collision energy (NCE) 26; maximum injection time 60 ms; dynamic exclusion time 50.0 s). The isolation window for MS/MS was set to 2.0 *m*/*z*.

### 4.9. Protein Identification and Label-Free Quantification (LFQ)

Proteins were identified using the Sequest HT algorithm implemented in Proteome Discoverer software (version 2.3, Thermo Fisher Scientific, Bremen, Germany). Carbamidomethylation of cysteines was selected as a fixed modification, whereas protein N-terminal acetylation, methionine oxidation, and phosphorylation were considered variable modifications. For tryptic peptides, a maximum of two missed cleavages was allowed. Precursor mass tolerance was set to 10 ppm, and the peptide and protein false discovery rate (FDR) was set at 0.01. The peptide identification sequence length was limited to 7–25 amino acids, and all other parameters were kept at their default settings. Peak lists were generated from raw data files using MaxQuant software (version 7.0.0, Max Planck Institute of Biochemistry, Martinsried, Germany) according to the standard workflow. This workflow corrects systematic errors in measured peptide masses and retention times of extracted peptides. Database searching was performed using the Sequest HT search engine (Thermo Fisher Scientific, Bremen, Germany) with the human UniProt database (version 2016) belonging to the Homo sapiens taxon.

### 4.10. Statistical Analysis of Differentially Expressed Proteins

Data analysis was performed using the open-access Perseus software (version 1.5.0, Max Planck Institute of Biochemistry, Martinsried, Germany). LFQ intensity values of proteins obtained from MaxQuant analysis were imported into Perseus and transformed to log_2_ values. Alpha error correction was performed using false discovery rate (FDR) correction defined by the Benjamini–Hochberg method.

### 4.11. Pathway, Molecular Function, and PPI Analysis

In the proteomic pathway analysis, differentially expressed proteins (DEPs) were evaluated using the PANTHER (Protein ANalysis Through Evolutionary Relationships) Classification System (University of Southern California, Los Angeles, CA, USA) and Reactome (Reactome Consortium, Hinxton, Cambridge, UK) bioinformatics tools to identify affected biological pathways. Protein–protein interaction (PPI) networks were constructed using the STRING (Search Tool for the Retrieval of Interacting Genes/Proteins) database (version 12.0; STRING Consortium, Zurich, Switzerland).

### 4.12. Integrated Functional Annotation of Differentially Expressed Proteins

Differentially expressed proteins identified in the groups were evaluated with an integrated, database-guided functional annotation procedure. Protein accession identifiers and gene symbols were standardized using UniProtKB records for Homo sapiens. Functional information for individual proteins was extracted from UniProtKB, Reactome pathway annotations, and Gene Ontology-associated biological descriptions. Theme assignment was based on documented protein functions and pathway associations rather than solely on protein-name keywords. Proteins with established roles in more than one biological process were assigned to multiple functional themes. Consequently, the cumulative number of theme assignments may exceed the total number of unique differentially expressed proteins. Functional themes were summarized by calculating the number and percentage of unique proteins assigned to each theme, together with the numbers of upregulated and downregulated proteins. Representative proteins were selected to illustrate the principal molecular components of each theme. The resulting classification was used to visualize the breadth of biological processes affected by drugs in single or combined applications. This procedure represents a descriptive, database-guided functional annotation and biological classification of the differentially expressed proteins, which was not performed as a Gene Ontology or KEGG over-representation analysis; hence, the presented Supplementary Table S3 did not include ratios or *p*-values of enrichments or false-discovery-rate-adjusted *p*-values. To facilitate visualization of the pathway diversity observed in the 72 h combination treatment group, the predominant pathways identified by the panther pathway classification were additionally summarized together with their associated protein counts and representative proteins (Supplementary Table S4).

## 5. Conclusions

Scientific reports of cell culture studies investigating candidate antineoplastic agents include thousands of examples in which the same agent was found to stimulate or inhibit cell growth even in the same cancer type. This fact is confusing for researchers seeking precise insights into a particular drug to predict whether the candidate drug could be clinically active and merit further development. Because numerous signaling pathways and countless individual molecules shape whether a drug exerts pro- or anticancer effects, comprehensive comparison of every detected pathway across studies reporting opposite results is nearly impossible. Instead, classifying these pathways as logically sound concepts would facilitate understanding why a drug leads to opposite biological outputs, enabling the design of better experiments and early clinical trials, and defining the target population who would benefit from this drug. This study investigated ATRA, curcumin, and their combination as potential agents for GBM. Both agents face brain entry limitations, but increasingly efficient nanoformulations may overcome these constraints. Furthermore, as both drugs cause much less local toxicity than classical chemotherapy agents, their combination can be safely injected into a GBM resection cavity using an Ommaya reservoir, which is used to deliver cytotoxic chemotherapy into brain tumors. Thus, understanding the mechanisms mediating hormesis versus anticancer synergistic effects of ATRA and curcumin may help match them to the tumors’ molecular pathological features and design patient-tailored treatments. The current study provides a humble contribution in these aspects.

### Limitations and Strengths

This study’s main limitation is the use of a single GBM cell line, which prevents broad generalization in view of the heterogeneity and complexity of GBM in patients. Validation in additional GBM cell lines, including patient-derived models, is therefore necessary to generalize the findings. Future study designs should include both spatial and temporal dimensions to better characterize the shift from hormesis to synergistic antineoplasticity. Using real-time impedance-based xCELLigence assays for proliferation and migration is a study strength, as they provide dynamic insights into cell behavior that are more informative than classical endpoint assays such as MTT, CCK-8, and scratch assays, which are mostly used in GBM cell culture studies.

## Figures and Tables

**Table 1:** Combination indices for All-trans retinoic acid (ATRA) and curcumin (CURC) cotreatments at different doses. Values were calculated with CompuSyn software and represent the results obtained at 72 h.

ATRA	Curcumin	FCA	CI
25 µM	5 µM	0.0748	299.722
25 µM	10 µM	0.0353	186.572
25 µM	20 µM	0.395	0.28584
50 µM	5 µM	0.0718	549.411
50 µM	10 µM	0.2393	0.50677
50 µM	20 µM	0.4870	0.17941
75 µM	5 µM	0.2669	0.31258
75 µM	10 µM	0.2523	0.51962
75 µM	20 µM	0.609	0.09409

Abbreviations: FCA, Fraction of Cells Affected; CI, Combination Index.

**Table 2:** Proteins showing differential expression across treatment groups. (Asteriks (*) indicates −log10(*p*) values.).

**Proteins with |Log2FC| Values ≥ 3 at Least in One Experimental Group**												
Protein Name (UniProt ID)	ATRA Log2FC				CURCUMIN Log2FC				COMBINATION Log2FC			
	−log10(*p*) *				−log10(*p*) *				−log10(*p*) *			
	48		72		48		72		48		72	
	DOWN	UP	DOWN	UP	DOWN	UP	DOWN	UP	DOWN	UP	DOWN	UP
SUN2 (Q9UH99): SUN domain-containing protein 2				3.296				2.240				
				4.735 *				4.370 *				
HXK2 (P52789): Hexokinase-2								1.347				3.303
								2.617 *				1.796 *
TGFBI (Q15582): Transforming growth factor-beta-induced protein ig-h3	−2.146		−4.229								−1.835	
	1.599 *		3.915 *								1.806 *	
TNC/Tenascin (P24821)	−2.332		−3.399									
	2.766 *		2.091 *									
HMOX1 (P09601): Heme oxygenase 1											−3.120	
											2.661 *	
**Proteins showing significant changes in all groups with |Log2FC| values ≥ 1.5**												
Protein Name (UniProt ID)	ATRA Log2FC				CURCUMIN Log2FC				COMBINATION Log2FC			
	−log10(*p*) *				−log10(*p*) *				−log10(*p*) *			
	48		72		48		72		48		72	
	DOWN	UP	DOWN	UP	DOWN	UP	DOWN	UP	DOWN	UP	DOWN	UP
UACA (Q9BZF9): Uveal autoantigen with coiled-coil domains and ankyrin repeats				1.936				1.454				1.794
				3.640 *				4.459 *				3.119 *
RPL37A (P61513): Large ribosomal subunit protein eL43		1.122				1.560				1.845		
		2.151 *				2.394 *				2.595 *		
TM9SF2 (Q99805): Transmembrane 9 superfamily member 2				1.514				1.240				2.056
				2.290 *				3.113 *				3.185 *
PHGDH/SERA (O43175): D-3-phosphoglycerate dehydrogenase	−1.63		−2.085								−1.219	
	3.031 *		5.898 *								1.979 *	
NUCB2 (P80303): Nucleobindin-2				−1.726			−1.015				−1.548	
				3.080 *			1.604 *				2.474 *	
SQSTM1 (Q13501): Sequestosome-1			−1.965				−1.214				−2.559	
			2.100 *				2.944 *				3.940 *	

## Data Availability

The data are not publicly available due to privacy. The data supporting the conclusions of this article will be made available by the corresponding author on reasonable request.

## Supplementary Materials

The following supporting information can be downloaded at: https://www.mdpi.com/article/10.3390/ijms27177795/s1.

## Abbreviations

The following abbreviations are used in this manuscript:

Akt	Protein kinase B
ANOVA	Analysis of variance
ATCC	American Type Culture Collection
ATRA	All-trans retinoic acid
Bax	Bcl-2-associated X protein
Bcl-2	B-cell lymphoma 2
BD	Becton Dickinson
CCK-8	Cell counting kit-8
CI	Combination index
CIM-Plate	Cell invasion and migration plate
CO_2_	Carbon dioxide
CompuSyn	Drug combination analysis software (ComboSyn Inc.)
CURC	Curcumin
DMEM	Dulbecco’s Modified Eagle’s Medium
DMSO	Dimethyl sulfoxide
ECM	Extracellular matrix
EMT	Epithelial–mesenchymal transition
ER	Endoplasmic reticulum
FBS	Fetal bovine serum
FITC	Fluorescein isothiocyanate
FlowJo	Flow cytometry data analysis software
GBM	Glioblastoma multiforme
GraphPad Prism	Biostatistics and graphing software
GSC	Glioblastoma stem cells
HDAC	Histone deacetylase
IC_50_	Half-maximal inhibitory concentration
IL	Interleukin
JAK/STAT	Janus kinase/signal transducer and activator of transcription
LC	Lower chamber
MAPK	Mitogen-activated protein kinase
MMP	Matrix metalloproteinase
MTT	3-(4,5-dimethylthiazol-2-yl)-2,5-diphenyl tetrazolium bromide assay
NF-κB	Nuclear factor kappa-light-chain-enhancer of activated B cells
Nrf2	Nuclear factor erythroid 2-related factor 2
NSC	Neural stem cell
OPC	Oligodendrocyte precursor cell
PBS	Phosphate-buffered saline
PI	Propidium iodide
PI3K	Phosphoinositide 3-kinase
RAR	Retinoic acid receptor
Rb/E2F	Retinoblastoma/E2F transcription factor pathway
ROS	Reactive oxygen species
RTCA	Real-time cell analyzer
RTCA Software	Real-time cell analyzer software (ACEA Biosciences/Agilent)
RUNX3	Runt-related transcription factor 3
SA-β-gal	Senescence-associated β-galactosidase
SD	Standard deviation
STAT3	Signal transducer and activator of transcription 3
TAB1	TGF-beta activated kinase 1 binding protein 1
TGF-β	Transforming growth factor beta
TMZ	Temozolomide
U87	Human glioblastoma cell line
UC	Upper chamber
VEGF	Vascular endothelial growth factor
WHO	World Health Organization
