# Supplementary material for: All-Trans Retinoic Acid and Curcumin Exhibit Hormesis or Synergistic Anticancer Effects in U87 Glioblastoma Cells: Defining the Proteome Accompanying Synergism
Source: Int J Mol Sci. 2026 Aug 31;27(17):7795. doi: 10.3390/ijms27177795 (PMC13566273; doi:10.3390/ijms27177795)
Supplement: Supplementary file 1 [file ijms-27-07795-s001.zip › ijms-4281879-supplementary.pdf]

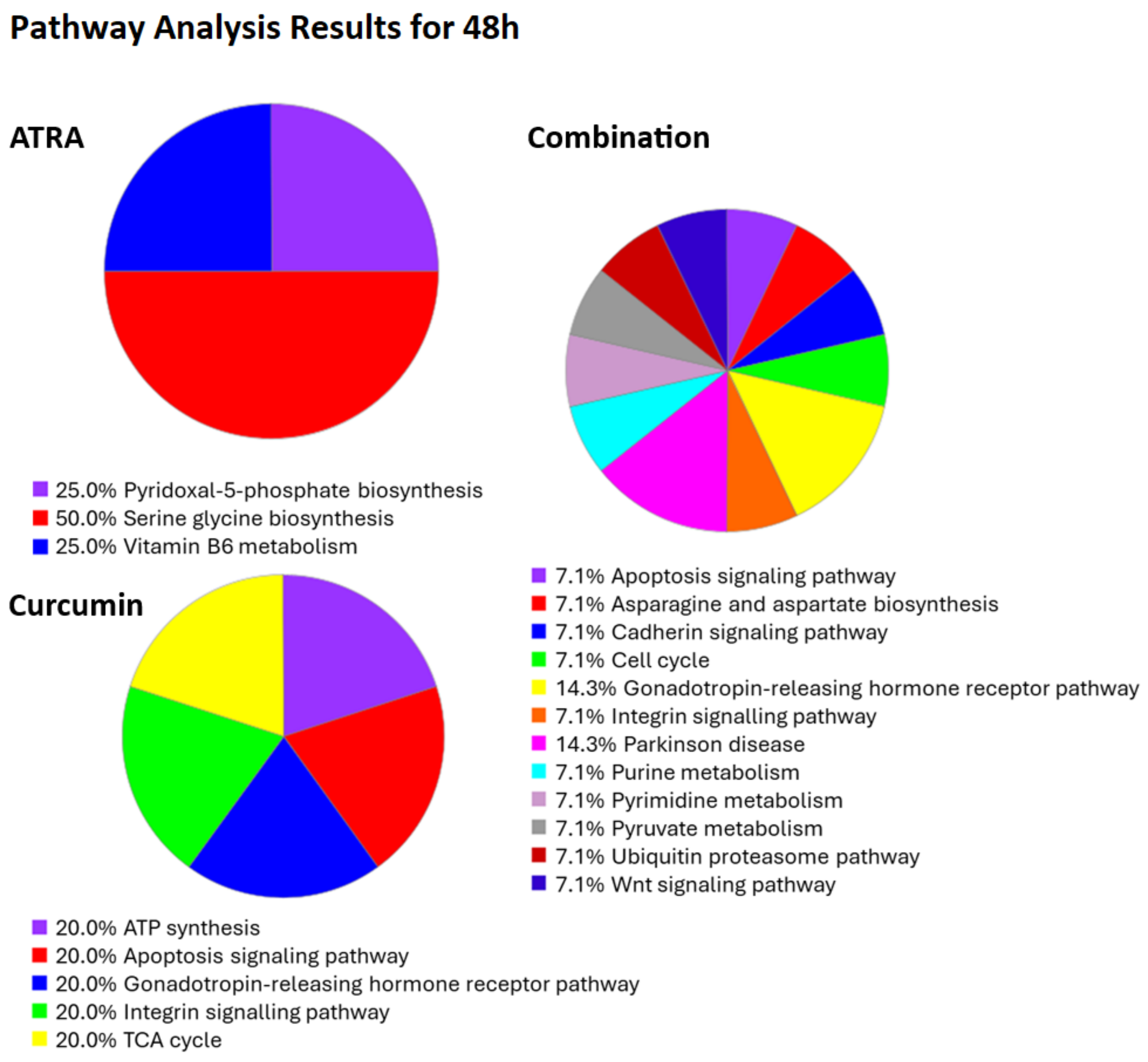

Supplementary Figure S1. Comparative pathway enrichment analysis of U87 glioblastoma cells treated with ATRA, Curcumin, and their combination at 48 hours.

The charts compare the biological pathways affected by ATRA, Curcumin, and their combination. While ATRA primarily targets amino acid and vitamin metabolism, Curcumin focuses on bioenergetics and apoptosis. The combination treatment significantly broadens the cellular response, activating diverse signaling networks including Wnt, Cell Cycle, and Ubiquitin-proteasome pathways, indicating a synergistic multi-target effect.

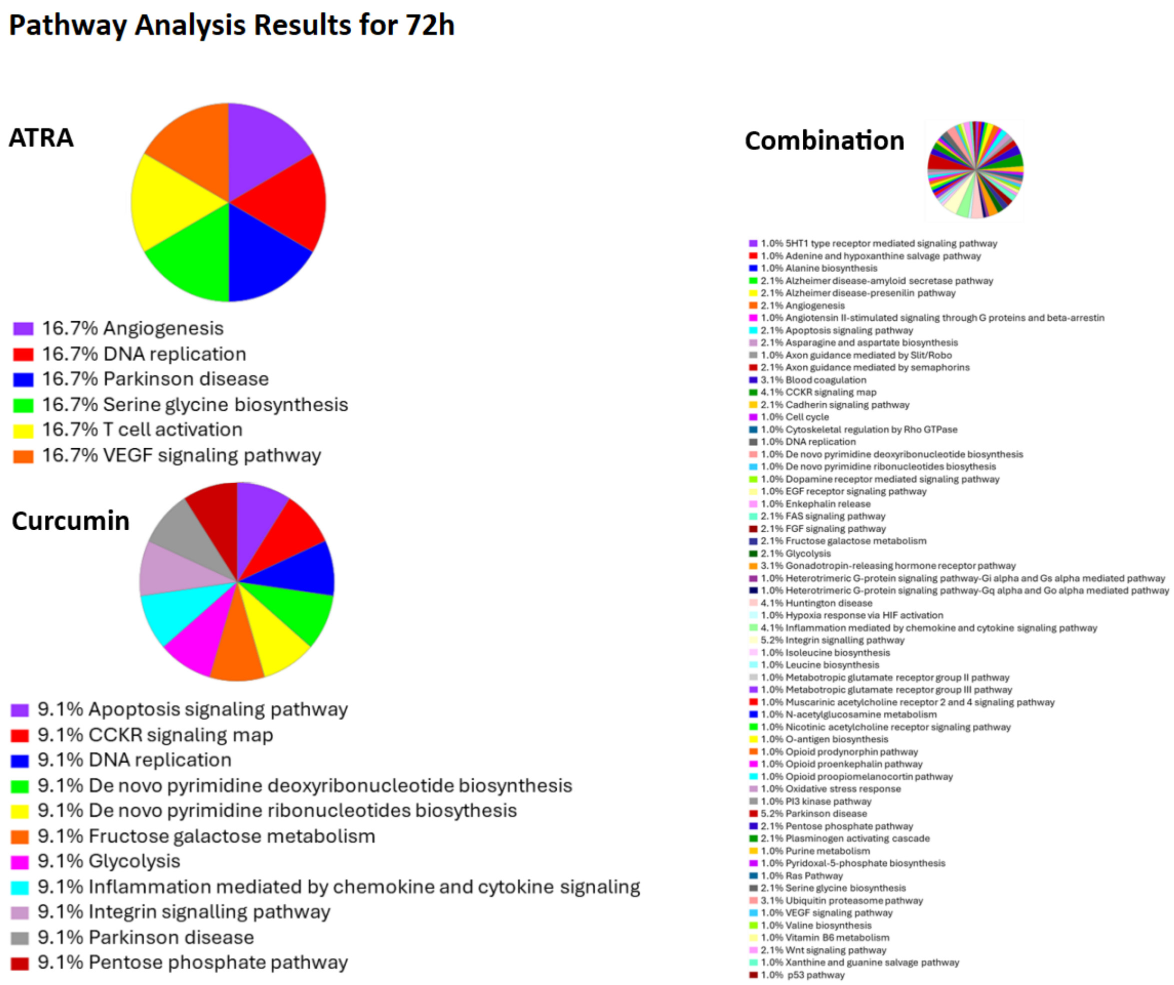

Supplementary Figure S2. Pathway enrichment analysis of U87 cells after 72h treatment.

At 72 hours, the molecular response shows increased complexity. ATRA treatment predominantly affects angiogenesis, VEGF signaling, and DNA replication. Curcumin demonstrates a broad impact on metabolic pathways (glycolysis, pentose phosphate) and inflammatory signaling. The combination group exhibits a highly diverse genomic profile, targeting a wide array of neurodegenerative pathways, cell cycle regulators, and advanced signaling cascades such as PI3K and p53, reflecting a robust and sustained synergistic effect over time.

Supplementary Table S1. Adjusted p-values from Sidak's multiple comparisons test of migraiton analysis. Results showed the effects of ATRA (75 μM), Curcumin (20 μM), and their combination compared to negative control at different time points (0–72 h). Significant differences are indicated as $p < 0.05$ for significant, ns: no significant. Data were obtained from xCELLigence real-time cell migration assays (n = 3 per group).

Time (h)	ATRA 75 μM	CURC 20 μM	ATRA 75 μM + CURC 20
0 h	ns	ns	ns
1 h	0.05	ns	0.05
2 h	0.05	ns	0.05
3 h	0.05	ns	0.05
4 h	0.05	ns	0.05
5 h	0.05	ns	0.05
6 h	0.05	ns	0.05
7 h	0.05	ns	0.05
8 h	0.05	ns	0.05
9 h	0.05	ns	0.05
10 h	0.05	ns	0.05
11 h	0.05	ns	0.05
12 h	0.05	ns	0.05
13 h	0.05	ns	0.05
14 h	0.05	ns	0.05
15 h	ns	ns	0.05
16 h	ns	ns	0.05
17 h	ns	ns	0.05
18 h	ns	ns	0.05
19 h	ns	ns	0.05
20 h	ns	ns	0.05

21 h	ns	ns	0.05
22 h	ns	ns	0.05
23 h	ns	ns	0.05
24 h	ns	ns	0.05
25 h	ns	ns	0.05
26 h	ns	ns	0.05
27 h	ns	ns	0.05
28 h	ns	ns	0.05
29 h	ns	ns	0.05
30 h	ns	ns	0.05
31 h	ns	ns	0.05
32 h	ns	ns	0.05
33 h	ns	ns	0.05
34 h	0.05	ns	0.05
35 h	0.05	ns	0.05
36 h	0.05	ns	0.05
37 h	0.05	ns	0.05
38 h	0.05	ns	0.05
39 h	0.05	0.05	0.05
40 h	0.05	0.05	0.05
41 h	0.05	0.05	0.05
42 h	0.05	0.05	0.05
43 h	0.05	0.05	0.05
44 h	0.05	0.05	0.05
45 h	0.05	0.05	0.05
46 h	0.05	0.05	0.05

47 h	0.05	0.05	0.05
48 h	0.05	0.05	0.05
49 h	0.05	0.05	0.05
50 h	0.05	0.05	0.05
51 h	0.05	0.05	0.05
52 h	0.05	0.05	0.05
53 h	0.05	0.05	0.05
54 h	0.05	0.05	0.05
55 h	0.05	0.05	0.05
56 h	0.05	0.05	0.05
57 h	0.05	0.05	0.05
58 h	0.05	0.05	0.05
59 h	0.05	0.05	0.05
60 h	0.05	0.05	0.05
61 h	0.05	0.05	0.05
62 h	0.05	0.05	0.05
63 h	0.05	0.05	0.05
64 h	0.05	0.05	0.05
65 h	0.05	0.05	0.05
66 h	0.05	0.05	0.05
67 h	0.05	0.05	0.05
68 h	0.05	0.05	0.05
69 h	0.05	0.05	0.05
70 h	0.05	0.05	0.05
71 h	0.05	0.05	0.05
72 h	0.05	0.05	0.05

Supplementary Table S2. The complete list of proteins showing statistically significant changes across all groups.

ATRA group results for 48h.

**Accession ID**	**Gene Name**	**Protein Name**	**-Log10(p)**	**Log2FC**
O43175	PHGDH	D-3-phosphoglycerate dehydrogenase	3,031	-1,630
P08195	SLC3A2	4F2 cell-surface antigen heavy chain	3,202	-2,215
P23381	WARS1	Tryptophan--tRNA ligase, cytoplasmic	2,830	-1,252
P24821	TNC	Tenascin	2,766	-2,332
P43307	SSR1	Translocon-associated protein subunit alpha	2,418	-1,050
P49591	SARS1	Serine--tRNA ligase, cytoplasmic	2,040	-1,237
Q15582	TGFBI	Transforming growth factor-beta-induced protein ig-h3	1,599	-2,146
Q92974	ARHGEF2	Rho guanine nucleotide exchange factor 2	1,942	-1,053
Q9Y617	PSAT1	Phosphoserine aminotransferase	2,498	-1,478
P18077	RPL35A	60S ribosomal protein L35a	2,830	1,879
Q6NVV1	RPL13AP3	Putative 60S ribosomal protein L13a protein RPL13AP3	2,117	1,201
P49736	MCM2	DNA replication licensing factor MCM2	3,002	1,144
P61513	RPL37A	60S ribosomal protein L37a	2,151	1,122

ATRA group results for 72h.

Accession ID	Gene Name	Protein Name	-Log10(p)	Log2FC
O43175	PHGDH	D-3-phosphoglycerate dehydrogenase	5,898	-2,085
O60711	LPXN	Leupaxin	3,103	-1,630
O95573	ACSL3	Long chain fattyacid CoA ligase 3	3,024	-3,870
P13674	P4HA1	Prolyl 4-hydroxylase subunit alpha-1	2,785	-1,085
P24821	TNC	Tenascin	2,091	-3,399
P30740	SERPINB1	Leukocyte elastase inhibitor	3,181	-1,185
P49257	LMAN1	Protein ERGIC-53	2,529	-1,456
P58546	MTPN	Myotrophin	2,276	-1,128
P61769	B2M	Beta-2-microglobulin	2,441	-1,285
P80303	NUCB2	Nucleobindin-2	3,080	-1,726
Q13162	PRDX4	Peroxiredoxin-4	2,456	-1,011
Q13501	SQSTM1	Sequestosome-1	2,100	-1,965
Q15582	TGFBI	Transforming growth factor-beta-induced protein ig-h3	3,915	-4,229
Q15843	NEDD8	Ubiquitin-like protein NEDD8	2,272	-1,241
Q92974	ARHGEF2	Rho guanine nucleotide exchange factor 2	3,430	-1,637
Q96AG4	LRRC59	Leucine-rich repeat-containing protein 59	2,548	-1,636
Q9NYL9	TMOD3	Tropomodulin-3	2,769	-1,185
O75915	ARL6IP5	PRA1 family protein 3	2,300	1,154
P05141	SLC25A5	ADP/ATP translocase 2	2,306	1,219
P33993	MCM7	DNA replication licensing factor MCM7	2,484	1,237
P55809	OXCT1	Succinyl-CoA:3-ketoacid coenzyme A transferase 1, mitochondrial	2,453	1,631
P62314	SNRPD1	Small nuclear ribonucleoprotein Sm D1	3,669	1,406
P84243	H3-3A	Histone H3.3	2,300	2,498
Q13616	CUL1	Cullin-1	2,430	1,220
Q27J81	INF2	Inverted formin-2	3,203	1,651
Q7L014	DDX46	Probable ATP-dependent RNA helicase DDX46	2,380	1,408
Q8NC51	SERBP1	Plasminogen activator inhibitor 1 RNA-binding protein	1,966	2,440
Q99805	TM9SF2	Transmembrane 9 superfamily member 2	2,290	1,514
Q9BZF9	UACA	Uveal autoantigen with coiled-coil domains and ankyrin repeats	3,640	1,936
Q9NVI7	ATAD3A	ATPase family AAA domain-containing protein 3A	2,112	1,484
Q9UH99	SUN2	SUN domain-containing protein 2	4,735	3,296

Curcumin group results for 48h.

Accession ID	Gene Name	Protein Name	-Log10 (p)	Log2FC
P04181	OAT	Ornithine aminotransferase, mitochondrial	2,609	-1,141
P53597	SUCLG1	Succinyl-CoA ligase [ADP/GDP-forming] subunit alpha, mitochondrial	2,562	-1,014
P99999	CYCS	Cytochrome c	2,355	-1,242
Q03135	CAV1	Caveolin-1	2,274	-1,094
Q8TCJ2	STT3B	Dolichyl-diphosphooligosaccharide--protein glycosyltransferase subunit STT3B	2,225	-1,077
Q92890	UFD1	Ubiquitin fusion degradation protein 1 homolog	1,991	-1,660
P60983	GMFB	Glia maturation factor beta	2,442	-1,126
O75821	EIF3G	Eukaryotic translation initiation factor 3 subunit G	2,300	1,187
P08865	RPSA	40S ribosomal protein SA	3,482	1,004
P61513	RPL37A	60S ribosomal protein L37a	2,394	1,560
P62826	RAN	GTP-binding nuclear protein Ran	2,302	1,908
Q06830	PRDX1	Peroxiredoxin-1	2,014	1,499
Q14247	CTTN	Src substrate cortactin	3,316	1,254
Q96HC4	PDLIM5	PDZ and LIM domain protein 5	2,355	1,206

Curcumin group results for 72h.

Accesion ID	Gene Name	Protein Name	$-Log_{10}$ (p)	Log_2FC
O60684	KPNA6	Importin subunit alpha-7	2,320	1,046
P12111	COL6A3	Collagen alpha-3(VI) chain	1,715	1,429
P18077	RPL35A	60S ribosomal protein L35a	2,587	1,059
P23919	DTYMK	Thymidylate kinase	2,005	1,270
P28340	POLD1	DNA polymerase delta catalytic subunit	1,883	1,245
P33992	MCM5	DNA replication licensing factor MCM5	2,391	1,362
P33993	MCM7	DNA replication licensing factor MCM7	3,294	1,264
P42285	MTREX	Superkiller viralicidic activity 2-like 2	2,702	1,455
P51572	BCAP31	B-cell receptor-associated protein 31	1,830	1,280
P52789	HK2	Hexokinase-2	2,617	1,347
P55809	OXCT1	Succinyl-CoA:3-ketoacid coenzyme A transferase 1	1,869	1,198
P61353	RPL27	60S ribosomal protein L27	1,977	1,720
P62244	RPS15A	40S ribosomal protein S15a	2,771	1,007
P84090	ERH	Enhancer of rudimentary homolog	1,546	1,396
Q07020	RPL18	60S ribosomal protein L18	3,268	1,096
Q14108	SCARB2	Lysosome membrane protein 2	1,687	1,083
Q16666	IFI16	Gamma-interferon-inducible protein 16	1,550	1,231
Q96CW1	AP2M1	AP-2 complex subunit mu	2,014	1,361
Q96P70	IPO9	Importin-9	1,927	1,283
Q99805	TM9SF2	Transmembrane 9 superfamily member 2	3,113	1,240
Q9BZF9	UACA	Uveal autoantigen with coiled-coil domains and ankyrin repeats	4,459	1,454
Q9UH99	SUN2	SUN domain-containing protein 2	4,370	2,240
Q9UJS0	SLC25A13	Calcium-binding mitochondrial carrier protein Aralar2	2,224	1,271
Q9UK76	JPT1	Hematological and neurological expressed 1 protein	1,888	1,129
B4DLN1	B4DLN1	Mitochondrial dicarboxylate carrier	2,043	1,030

ATRA-Curcumin combination group results for 48h.

Accession ID	Gene Name	Protein Name	-Log10 (p)	Log2FC
P62308	SNRPG	Small nuclear ribonucleoprotein G	2,524	-1,320
O00232	PSMD12	26S proteasome non-ATPase regulatory subunit 12	2,259	-1,117
O75531	BANF1	Barrier-to-autointegration factor	1,982	-1,192
P00441	SOD1	Superoxide dismutase [Cu-Zn]	3,185	-1,179
P08195	SLC3A2	Amino acid transporter heavy chain SLC3A2	2,194	-1,242
P08962	CD63	CD63 antigen	1,670	-1,103
P09211	GSTP1	Glutathione S-transferase P	5,177	-1,166
P0DMV9	HSPA1B	Heat shock 70 kDa protein 1B	5,225	-1,648
P17174	GOT1	Aspartate aminotransferase, cytoplasmic	3,885	-1,428
P22061	PCMT1	Protein-L-isoaspartate(D-aspartate) O-methyltransferase	3,090	-1,372
P36543	ATP6V1E1	V-type proton ATPase subunit E 1	1,936	-1,043
P40926	MDH2	Malate dehydrogenase, mitochondrial	2,144	-1,085
P48723	HSPA13	Heat shock 70 kDa protein 13	2,158	-1,073
P49773	HINT1	Adenosine 5'-monophosphoramidase HINT1	1,863	-1,043
P51116	FXR2	RNA-binding protein FXR2	2,824	-1,308
P55209	NAP1L1	Nucleosome assembly protein 1-like 1	3,165	-1,093
P61960	UFM1	Ubiquitin-fold modifier 1	1,976	-1,421
P67870	CSNK2B	Casein kinase II subunit beta	1,631	-1,832
P78417	GSTO1	Glutathione S-transferase omega-1	1,432	-1,517
Q03135	CAV1	Caveolin-1	2,999	-1,412
Q04828	AKR1C1	Aldo-keto reductase family 1 member C1	1,837	-1,603
Q13724	MOGS	Mannosyl-oligosaccharide glucosidase	3,064	-1,076
Q16881	TXNRD1	Thioredoxin reductase 1, cytoplasmic	2,256	-1,082
Q96AG4	LRRC59	Leucine-rich repeat-containing protein 59	2,151	-1,238
Q96HY6	DDRGK1	DDRGK domain-containing protein 1	2,999	-1,843
Q96M27	PRRC1	Protein PRRC1	3,118	-1,430
O00148	DDX39A	ATP-dependent RNA helicase DDX39A	2,251	1,770

P06753	TPM3	Tropomyosin alpha-3 chain	1,604	1,218
P13489	RNH1	Ribonuclease inhibitor	2,010	1,038
P18621	RPL17	Large ribosomal subunit protein uL22	2,088	1,273
P21589	NT5E	5'-nucleotidase	3,154	1,378
P23526	AHCY	Adenosylhomocysteinase	1,945	1,099
P34932	HSPA4	Heat shock 70 kDa protein 4	1,901	1,219
P36578	RPL4	Large ribosomal subunit protein uL4	3,138	2,291
P36871	PGM1	Phosphoglucomutase-1	1,985	1,244
P49736	MCM2	DNA replication licensing factor MCM2	2,678	1,103
P53618	COPB1	Coatomer subunit beta	2,567	1,886
P61513	RPL37A	Large ribosomal subunit protein eL43	2,595	1,845
P62277	RPS13	Small ribosomal subunit protein uS15	2,518	1,062
P62805	H4C1	Histone H4	1,668	1,444
P62913	RPL11	Large ribosomal subunit protein uL5	1,870	1,419
Q8IVF2	AHNAK2	Protein AHNAK2	2,258	1,090
Q92688	ANP32B	Acidic leucine-rich nuclear phosphoprotein 32 family member B	3,102	2,562
Q9UJS0	SLC25A13	Electrogenic aspartate/glutamate antiporter SLC25A13, mitochondrial	2,325	1,437

ATRA-Curcumin combination group results for 72h.

Accesion ID	Gene Name	Protein Name	-Log10 (p)	Log2FC
O43175	PHGDH	D-3-phosphoglycerate dehydrogenase	1,979	-1,125
O43684	BUB3	Mitotic checkpoint protein BUB3	1,988	-1,165
O60711	LPXN	Leupaxin	2,012	-1,225
O60739	EIF1B	Eukaryotic translation initiation factor 1b	2,569	-1,037
O75531	BANF1	Barrier-to-autointegration factor	2,518	-1,037
O75934	BCAS2	Pre-mRNA-splicing factor SPF27	2,591	-1,003
O96019	ACTL6A	Actin-like protein 6A	2,287	-1,152
P00441	SOD1	Superoxide dismutase [Cu-Zn]	1,869	-1,556
P00491	PNP	Purine nucleoside phosphorylase	2,047	-1,041
P05067	APP	Amyloid beta A4 protein	3,793	-2,987
P05120	SERPINB2	Plasminogen activator inhibitor 2	1,907	-1,130
P05121	SERPINE1	Plasminogen activator inhibitor 1	2,233	-2,353
P08243	ASNS	Asparagine synthetase [glutamine-hydrolyzing]	1,277	-1,527
P09601	HMOX1	Heme oxygenase 1	2,661	-3,120
P09936	UCHL1	Ubiquitin carboxyl-terminal hydrolase isozyme L1	2,328	-1,027
Q71UI9	H2AZ2	Histone H2A.V	1,424	-1,007
P0DMV8	HSPA1A	Heat shock 70 kDa protein 1A	2,715	-1,462
P10599	TXN	Thioredoxin	1,858	-1,181
P11172	UMPS	Uridine 5-monophosphate synthase	2,650	-1,730
P14174	MIF	Macrophage migration inhibitory factor	2,069	-1,061
P17174	GOT1	Aspartate aminotransferase, cytoplasmic	2,909	-1,531
P17980	PSMC3	26S protease regulatory subunit 6A	3,087	-1,124
P20618	PSMB1	Proteasome subunit beta type-1	3,101	-1,215
P20674	COX5A	Cytochrome c oxidase subunit 5A, mitochondrial	2,059	-1,430
P21281	ATP6V1B2	V-type proton ATPase subunit B, brain isoform	2,635	-1,317
P22061	PCMT1	Protein-L-isoaspartate(D-aspartate) O-methyltransferase	1,726	-1,219
P23381	WARS1	Tryptophan--tRNA ligase, cytoplasmic	3,374	-1,499
P30043	BLVRB	Flavin reductase (NADPH)	4,177	-1,683
P30044	PRDX5	Peroxiredoxin-5, mitochondrial	1,267	-1,250
P30048	PRDX3	Thioredoxin-dependent peroxide reductase, mitochondrial	2,684	-1,299
P30086	PEBP1	Phosphatidylethanolamine-binding protein 1	3,934	-1,134
P35754	GLRX	Glutaredoxin-1	2,309	-1,431
P41250	GARS1	Glycine--tRNA ligase	3,884	-1,685
P51991	HNRNPA3	Heterogeneous nuclear ribonucleoprotein A3	2,329	-1,103
P54687	BCAT1	Branched-chain-amino-acid aminotransferase, cytosolic	2,126	-1,049
P55209	NAP1L1	Nucleosome assembly protein 1-like 1	2,151	-1,422
P62333	PSMC6	26S protease regulatory subunit 10B	2,399	-1,109
P62899	RPL31	60S ribosomal protein L31	1,291	-1,300
P63151	PPP2R2A	Serine/threonine-protein phosphatase 2A 55 kDa regulatory subunit B alpha isoform	1,654	-1,319

P68402	PAFAH1B2	Platelet-activating factor acetylhydrolase IB subunit beta	2,378	-1,210
P80303	NUCB2	Nucleobindin-2	2,474	-1,548
Q04828	AKR1C1	Aldo-keto reductase family 1 member C1	2,958	-1,953
Q08379	GOLGA2	Golgin subfamily A member 2	1,305	-1,578
Q13162	PRDX4	Peroxiredoxin-4	3,397	-1,236
Q13501	SQSTM1	Sequestosome-1	3,940	-2,559
Q13561	DCTN2	Dynactin subunit 2	3,157	-1,287
Q13951	CBFB	Core-binding factor subunit beta	1,663	-1,291
Q14847	LASP1	LIM and SH3 domain protein 1	2,066	-1,334
Q15582	TGFBI	Transforming growth factor-beta-induced protein ig-h3	1,806	-1,835
Q15758	SLC1A5	Neutral amino acid transporter B(0)	2,853	-1,328
Q16643	DBN1	Drebrin	1,317	-1,652
Q16881	TXNRD1	Thioredoxin reductase 1, cytoplasmic	3,095	-1,439
Q8NBS9	TXNDC5	Thioredoxin domain-containing protein 5	2,473	-1,079
Q9BU89	DOHH	Deoxyhypusine hydroxylase	4,724	-1,157
Q9H299	SH3BGRL3	SH3 domain-binding glutamic acid-rich-like protein 3	2,685	-1,171
Q9NR45	NANS	Sialic acid synthase	1,722	-1,478
Q9NUJ1	ABHD10	Mycophenolic acid acyl-glucuronide esterase, mitochondrial	2,237	-1,345
Q9UI30	TRMT112	Multifunctional methyltransferase subunit TRM112-like protein	2,238	-1,203
Q9UI47	CTNNA3	Catenin alpha-3	1,134	-2,702
Q9UJ70	NAGK	N-acetyl-D-glucosamine kinase	1,438	-1,144
Q9Y224	RTRAF	UPF0568 protein C14orf166	3,037	-1,077
Q9Y2W1	THRAP3	Thyroid hormone receptor-associated protein 3	1,821	-1,416
Q9Y617	PSAT1	Phosphoserine aminotransferase	3,240	-1,605
O00232	PSMD12	26S proteasome non-ATPase regulatory subunit 12	2,762	1,267
O00410	IPO5	Importin-5	1,640	1,185
O14786	NRP1	Neuropilin-1	3,082	1,526
O14980	XPO1	Exportin-1	1,447	1,421
O43143	DHX15	Pre-mRNA-splicing factor ATP-dependent RNA helicase DHX15	1,885	1,644
O43390	HNRNPR	Heterogeneous nuclear ribonucleoprotein R	3,034	1,036
O43847	NRDC	Nardilysin	1,112	1,375
O60684	KPNA6	Importin subunit alpha-7	2,185	1,443
O75533	SF3B1	Splicing factor 3B subunit 1	3,415	1,088
O75915	ARL6IP5	PRA1 family protein 3	1,452	2,055
O94808	GFPT2	Glutamine--fructose-6-phosphate aminotransferase [isomerizing] 2	2,908	1,105
P04844	RPN2	Dolichyl-diphosphooligosaccharide--protein glycosyltransferase subunit 2	2,583	1,319
P04899	GNAI2	Guanine nucleotide-binding protein G(i) subunit alpha-2	2,858	1,128
P05023	ATP1A1	Sodium/potassium-transporting ATPase subunit alpha-1	2,364	1,067
P05141	SLC25A5	ADP/ATP translocase 2	3,334	1,652
P05556	ITGB1	Integrin beta-1	4,409	1,147
P06703	ATP5F1B	Protein S100-A6	3,607	1,195
P07384	CAPN1	Calpain-1 catalytic subunit	2,935	1,532

P08134	RHOC	Rho-related GTP-binding protein RhoC	1,160	1,195
P08708	RPS17	40S ribosomal protein S17	1,494	1,399
P12111	COL6A3	Collagen alpha-3(VI) chain	2,157	1,842
P15880	RPS2	40S ribosomal protein S2	3,571	1,096
P17655	CAPN2	Calpain-2 catalytic subunit	1,617	1,105
P18077	RPL35A	60S ribosomal protein L35a	4,347	1,999
P18754	RCC1	Regulator of chromosome condensation	2,297	1,051
P19367	HK1	Hexokinase-1	2,330	1,295
P20700	LMNB1	Lamin-B1	1,902	1,034
P20962	PTMS	Parathymosin	2,272	1,413
P23396	RPS3	40S ribosomal protein S3	3,015	1,009
P23526	AHCY	Adenosylhomocysteinase	2,342	1,396
P23919	DTYMK	Thymidylate kinase	2,195	1,536
P26006	ITGA3	Integrin alpha-3	1,245	1,382
P27695	APEX1	DNA-(apurinic or apyrimidinic site) lyase	1,185	1,218
P33992	MCM5	DNA replication licensing factor MCM5	3,270	1,948
P33993	MCM7	DNA replication licensing factor MCM7	4,194	1,572
P35221	CTNNA1	Catenin alpha-1	2,993	1,015
P36578	RPL4	60S ribosomal protein L4	2,810	1,069
P39656	DDOST	Dolichyl-diphosphooligosaccharide--protein glycosyltransferase 48 kDa subunit	1,370	1,133
P40429	RPL13A	60S ribosomal protein L13a	1,745	1,105
P40616	ARL1	ADP-ribosylation factor-like protein 1	1,713	1,306
P42285	MTREX	Superkiller viralicidic activity 2-like 2	2,354	2,174
P42704	LRPPRC	Leucine-rich PPR motif-containing protein, mitochondrial	3,857	1,563
P46776	RPL27A	60S ribosomal protein L27a	2,280	1,570
P49736	MCM2	DNA replication licensing factor MCM2	2,817	1,007
P51572	BCAP31	B-cell receptor-associated protein 31	1,792	2,112
P52789	HK2	Hexokinase-2	1,796	3,303
P53621	COPA	Coatomer subunit alpha	1,473	1,074
P54727	RAD23B	UV excision repair protein RAD23 homolog B	1,135	1,057
P55060	CSE1L	Exportin-2	1,056	1,824
P55809	OXCT1	Succinyl-CoA:3-ketoacid coenzyme A transferase 1, mitochondrial	3,759	1,436
P61353	RPL27	60S ribosomal protein L27	2,889	2,435
P62244	RPS15A	40S ribosomal protein S15a	2,252	1,193
P62424	RPL7A	60S ribosomal protein L7a	2,530	1,566
P62805	H4C16	Histone H4	4,001	1,277
P62906	RPL10A	60S ribosomal protein L10a	3,479	2,150
P62910	RPL32	60S ribosomal protein L32	1,769	1,155
P62913	RPL11	60S ribosomal protein L11	2,421	1,411
P63010	AP2B1	AP-2 complex subunit beta	2,199	1,462
P78527	PRKDC	DNA-dependent protein kinase catalytic subunit	1,753	1,417
P84090	ERH	Enhancer of rudimentary homolog	1,612	1,489
Q02543	RPL18A	60S ribosomal protein L18a	1,705	2,289
Q07020	RPL18	60S ribosomal protein L18	4,089	1,819
Q07666	KHDRBS1	KH domain-containing, RNA-binding, signal transduction-associated protein 1	2,121	1,259
Q12965	MYO1E	Unconventional myosin-Ie	1,745	1,219

Q13616	CUL1	Cullin-1	4,513	1,245
Q14108	SCARB2	Lysosome membrane protein 2	2,655	1,278
Q15363	TMED2	Transmembrane emp24 domain-containing protein 2	2,193	1,070
Q16531	DDB1	DNA damage-binding protein 1	4,059	1,344
Q16666	IFI16	Gamma-interferon-inducible protein 16	2,897	1,220
Q16795	NDUFA9	NADH dehydrogenase [ubiquinone] 1 alpha subcomplex subunit 9, mitochondrial	2,042	1,203
Q27J81	INF2	Inverted formin-2	3,137	1,731
Q6P2Q9	PRPF8	Pre-mRNA-processing-splicing factor 8	4,290	1,174
Q6PIU2	NCEH1	Neutral cholesterol ester hydrolase 1	1,479	1,282
Q71DI3	H3C15; H3C14; H3C13	Histone H3.2	1,586	1,286
Q7L1Q6	BZW1	Basic leucine zipper and W2 domain-containing protein 1	2,823	1,023
Q7Z6Z7	HUWE1	E3 ubiquitin-protein ligase HUWE1	1,333	1,068
Q86X55	CARM1	Histone-arginine methyltransferase CARM1	1,593	1,354
Q8TED1	GPX8	Probable glutathione peroxidase 8	1,979	1,323
Q8TEX9	IPO4	Importin-4	2,042	1,411
Q96AB3	ISOC2	Isochorismatase domain-containing protein 2, mitochondrial	1,733	1,573
Q96C90	PPP1R14B	Protein phosphatase 1 regulatory subunit 14B	2,496	1,219
Q96CW1	AP2M1	AP-2 complex subunit mu	2,336	1,636
Q99460	PSMD1	26S proteasome non-ATPase regulatory subunit 1	4,455	1,103
Q99805	TM9SF2	Transmembrane 9 superfamily member 2	3,185	2,056
Q9BPX3	NCAPG	Condensin complex subunit 3	1,782	1,208
Q9BZF9	UACA	Uveal autoantigen with coiled-coil domains and ankyrin repeats	3,119	1,794
Q9H3U1	UNC45A	Protein unc-45 homolog A	1,329	1,116
Q9HCJ6	VAT1L	Synaptic vesicle membrane protein VAT-1 homolog-like	1,850	1,101
Q9NYU2	UGGT1	UDP-glucose:glycoprotein glucosyltransferase 1	2,667	1,662
Q9UJS0	SLC25A13	Calcium-binding mitochondrial carrier protein Aralar2	3,426	1,750
Q9UKK3	PARP4	Poly [ADP-ribose] polymerase 4	2,453	1,602
Q9UNS2	COPS3	COP9 signalosome complex subunit 3	1,286	1,158
B4DLN1	B4DLN1	Mitochondrial dicarboxylate carrier	1,127	1,409

Supplementary Table S3. Integrated Functional Annotation of Differentially Expressed Proteins Following Combined ATRA–Curcumin Treatment (72 h)

Functional Theme	Total Proteins	Down regulated	Up regulated	Representative Proteins	Biological Interpretation

RNA Processing, Splicing and Translation	33	6	27	SF3B1, PRPF8, DHX15, BCAS2, HNRNPR	Extensive remodeling of RNA maturation, splicing and translational machinery.
Metabolism (Amino Acid, Nucleotide and Carbohydrate)	23	11	12	PHGDH, PSAT1, HK2, HK1, GOT1, BCAT1	Broad metabolic reprogramming affecting biosynthetic and energetic pathways.
Cell Adhesion, ECM and Cytoskeletal Organization	21	10	11	ITGA3, ITGB1, COL6A3, TGFBI, CTNNA1	Altered extracellular matrix interactions and structural remodeling.
Mitochondrial Function and Energy Metabolism	20	9	11	COX5A, ATP5F1B, OXCT1, SLC25A13	Reorganization of mitochondrial bioenergetics and metabolite transport.
Stress Response, Apoptosis and Immune Regulation	20	13	7	HMOX1, APP, MIF, IFI16, HSPA1A	Coordinated cellular stress response accompanying treatment.
DNA Replication, Repair and Chromosome Organization	19	5	14	MCM2, MCM5, MCM7, DDB1, PRKDC	Activation of genome maintenance and proliferative control pathways.
Redox Homeostasis and Oxidative Stress	14	13	1	TXN, TXNRD1, PRDX3, PRDX4, GLRX	Marked remodeling of antioxidant defense systems.
Proteasome, Ubiquitin and Protein Quality Control	14	8	6	PSMB1, PSMC3, PSMD1, PSMD12, SQSTM1	Altered proteostasis and ubiquitin-dependent protein turnover.

Supplementary Table S4. Predominant panther pathway classifications identified in the 72 h combination treatment group and their representative proteins.

Pathway	Percentage of identified pathways	Protein count	Representative proteins
Integrin signaling pathway	5.2%	5	ITGB1, ITGA3, RHOC, COL6A3, ARL1
Parkinson disease pathway	5.2%	5	MCM5, UCHL1, CUL1, PSMB1, HSPA1A

Huntington disease pathway	4.1%	4	CAPN1, CAPN2, ARL1, VAT1L
Chemokine and cytokine-mediated inflammation	4.1%	4	ITGB1, COL6A3, GNAI2, RHOC
CCKR signaling map	4.1%	4	SERPINB2, ITGB1, SERBP1, AP2M1
